# Incidence of Dementia in Older Adults Hospitalized With Acute Coronary Syndrome: Insights From KP-Brain-Heart Health Study

**DOI:** 10.1016/j.jacadv.2026.103192

**Published:** 2026-08-28

**Authors:** Ashok Krishnaswami, Amanda Jain, Paola Gilsanz, Wynnelena Canio, Richard Ha, Deepak L. Bhatt, Mathew S. Maurer, Michael G. Nanna, Neelum T. Aggarwal, Michelle M. Mielke, Annabelle S. Volgman, Howard Dinh, Janine Y. Yang, Edward D. Shin, Abdulla A. Damluji, Ahmed I. Shah

**Affiliations:** a The Permanente Medical Group, Division of Cardiology, Kaiser Permanente San Jose Medical Center, San Jose, California, USA; b Kaiser Permanente, Division of Research, Pleasanton, California, USA; c The Permanente Medical Group, Department of Geriatrics, Kaiser Permanente San Rafael Medical Center, San Rafael, California, USA; d The Permanente Medical Group, Department of Cardiovascular Surgery, Kaiser Permanente Santa Clara Medical Center, Santa Clara, California, USA; e Mount Sinai Fuster Heart Hospital, Icahn School of Medicine at Mount Sinai, New York, New York, USA; f Division of Cardiology, Columbia University Irving Medical Center, New York, New York, USA; g Section of Cardiovascular Medicine, Yale School of Medicine, New Haven, Connecticut, USA; h Department of Neurological Sciences, Rush Alzheimer’s Disease Center, Chicago, Illinois, USA; i Department of Epidemiology and Prevention, Public Health Sciences, Wake Forest University, School of Medicine, Winston-Salem, North Carolina, USA; j Division of Cardiology, Rush University Medical Center, Chicago, Illinois, USA; k The Permanente Medical Group, Department of Cardiology, Kaiser Permanente South Sacramento Medical Center, South Sacramento, California, USA; l Department of Internal Medicine, Kaiser Permanente, Santa Clara Medical Center, Santa Clara, California, USA; m Department of Internal Medicine, Kaiser Permanente, Oakland Medical Center; Oakland, California, USA; n Cardiovascular Center on Aging, Department of Cardiovascular Medicine, Cleveland Clinic Foundation, Cleveland, Ohio, USA; o The Permanente Medical Group, Department of Cardiology, Kaiser Permanente Oakland Medical Center, Oakland, California, USA

**Keywords:** acute coronary syndrome, cognitive function, coronary artery bypass grafting, dementia incidence, percutaneous coronary intervention, stable coronary artery disease

## Abstract

**Background:**

Cognitive decline has been reported after coronary artery revascularization, but whether it reflects procedure-specific effects or the burden of underlying vascular disease remains uncertain.

**Objectives:**

To determine whether incident dementia risk differs according to coronary revascularization strategy among older adults with acute coronary syndrome (ACS).

**Methods:**

We conducted a longitudinal cohort study among adults aged ≥65 years hospitalized for ACS between 2010 and 2020 (n = 25,176). Patients underwent percutaneous coronary intervention (n = 8,043), coronary artery bypass grafting (n = 797), or received no revascularization (n = 16,336). A second comparator cohort included patients with stable coronary artery disease (CAD) without revascularization (n = 154,299). The primary outcome was incident dementia identified using validated International Classification of Diseases codes after a 1-year washout. Propensity-matched analyses used Cox models accounting for the competing risk of death.

**Results:**

Revascularized patients were younger (74.8 ± 6.9 vs 77.1 ± 8.2 years), more often male (66.2% vs 54.0%), and had lower Elixhauser multimorbidity scores (4.6 ± 2.8 vs 5.4 ± 3.0, all *P* < 0.001). Over a median 4.8 years of follow-up, 9.0% of ACS patients developed dementia. Revascularization was not associated with increased dementia risk compared with ACS without revascularization (sub-hazard ratio: 1.05; 95% CI: 0.95-1.17) or stable CAD. Dementia risk was also similar between percutaneous coronary intervention and coronary artery bypass grafting.

**Conclusions:**

We found that among older adults with ACS, coronary revascularization was not statistically significantly associated with increased dementia risk compared with no revascularization or stable CAD. These findings reflect the hypothesis that the majority of dementia risk is potentially driven more by cumulative vascular and systemic factors than by procedure-specific neurotoxicity.

Cognitive decline and dementia are increasingly recognized as major determinants of quality of life and independence in older adults with cardiovascular disease.^1^ Concerns that coronary revascularization may accelerate cognitive impairment have persisted, particularly in the context of cardiopulmonary bypass or perioperative embolic phenomena. However, systematic reviews of randomized trials have shown no long-term cognitive difference between off-pump and on-pump coronary artery bypass grafting (CABG),^2^, ^3^, ^4^ and observational data indicate that cognitive function declines similarly over time following CABG and percutaneous coronary revascularization (PCI).^3^, ^4^, ^5^

Despite these findings, uncertainty remains^6^ whether coronary revascularization in the setting of acute coronary syndrome (ACS) contributes to long-term dementia risk^7^ or merely reflects the cumulative burden of vascular disease, inflammation, and aging.^8^ Previous studies have often been limited by small sample sizes, short follow-up periods, lack of comparator group, or failure to account for competing risks of death in older populations. Moreover, whether cognitive outcomes after revascularization reflect procedure-specific neurotoxicity or the broader vascular and systemic components is unclear.^7^

To address these gaps, we conducted a large retrospective cohort study within Kaiser Permanente Northern California (KPNC), an integrated healthcare-delivery system with comprehensive longitudinal data. We examined older adults (≥65 years) hospitalized with ACS who underwent PCI or CABG and compared them with those managed medically, followed by a second comparison with those with stable coronary artery disease (CAD). Recognizing the high competing risk of death in this population, we applied competing-risk models, an approach not used in previous studies, to determine whether coronary revascularization overall and specific procedural type were independently associated with long-term dementia risk. The null hypothesis was that incident dementia with coronary revascularization would be equivalent to ACS, no revascularization, and to stable CAD.

## Methods

### Source population

The source population was from KPNC, a large, integrated healthcare-delivery system providing comprehensive medical care to approximately 4.6 million insured individuals across the Northern California region.^9^ Patients insured by Kaiser Permanente are demographically and socioeconomically similar to other insured population in the catchment area. However, KPNC-insured patients have a lower proportion of individuals at the lower income tail.^10^ The healthcare-delivery system comprises of 25 free-standing medical clinics and 21 hospitals of which 12 have PCI capability and 3 are capable of CABG and other cardiovascular surgical procedures. These facilities operate within a coordinated hub-and-spoke model, supported by standardized interfacility transfer protocols that ensure timely and equitable access to specialized cardiac services across the region.

#### Study Cohort

This study represents part of the overall KPNC Brain-Heart Health Study_IHD_ (in Ischemic Heart Disease)—a longitudinal research program designed to evaluate cognitive outcomes and determinants of brain-heart health among older adults with ischemic heart disease syndromes. The present investigation represents the second study from this cohort. We have previously reported on baseline prevalence of all-cause and subtypes of dementia in this cohort.^11^ The current study was a retrospective cohort study of adults aged ≥65 years who were hospitalized for ACS between January 1, 2010, and December 31, 2020. Data were obtained from the comprehensive KPNC electronic health record system (EPIC Systems), which captures demographics, diagnoses, procedures, laboratory results, and medication records across care settings. Information on the primary hospital discharge diagnosis of ACS was obtained using the International Classification of Diseases*-*9th Revision (ICD-9) codes and ICD-10 codes based on the year of admission^12^ (Supplemental Table 1). An ACS diagnosis was determined by hospitalists and was defined as acute chest pain or equivalent ischemic symptoms in association with either elevated cardiac biomarkers or new ischemic changes on electrocardiography, including dynamic ST-segment or T-wave abnormalities. The flow diagram of patient entry into the study is shown in Supplemental Figure 1.

#### Primary exposure

The primary exposure was defined as undergoing coronary revascularization (PCI or CABG). Identification of patients who underwent a PCI or CABG and procedural details was supplemented using the KPNC CEDARON Cardiac Care database and the Society of Thoracic Surgeons toolkit which contains standardized data on cardiac catheterization and coronary revascularization. The PCI cohort included all patients including those who underwent multivessel or left-main percutaneous coronary intervention using second- or higher-generation drug-eluting stents. The CABG cohort consisted of patients who underwent isolated CABG without concomitant valve or aortic procedures. We excluded patients if they had prior CABG, cardiogenic shock, or cardiac arrest within 24 hours of admission. Index revascularization was defined as the first procedure during the qualifying hospitalization for ACS.

The nonrevascularized group consisted of patients who had an index hospitalization with an ACS diagnosis and who did not undergo PCI or CABG. This group included individuals with low-level troponin elevations or alternative final clinical diagnoses (eg, myocarditis, pericarditis, myopericarditis, or type 2 myocardial infarction), as well as patients managed conservatively with medical therapy, noninvasive testing (eg, stress testing), or diagnostic coronary angiography without revascularization based on intraprocedural assessments. To ensure accurate classification of ACS, coronary revascularization status, and stable CAD, we performed a direct chart review for additional validation. This review included a subset of patients who underwent coronary revascularization (n = 50) and those with ACS who did not undergo revascularization (n = 50).

### Primary outcome

The primary outcome was incident all-cause dementia, and dementia subtypes, identified from electronic medical records using ICD-9/10 codes (Supplemental Table 2) and validated by structured chart review. Patients with documented dementia before index hospitalization or within 1 year after the index hospitalization were excluded. A subset of patients with dementia (n = 100) underwent additional validation by direct chart review to ensure diagnostic accuracy of the presence of all-cause dementia.

### Covariates

The baseline covariates used in this study included patient demographics (age at study entry, sex, race/ethnicity, body mass index, smoking status, neighborhood deprivation index), multimorbidity burden (Elixhauser comorbidity index), individual comorbidities (diabetes mellitus, hypertension, hyperlipidemia, cerebrovascular disease, peripheral vascular disease, heart failure, valvular heart disease, atrial fibrillation, malignancy, anemia, chronic lung disease, sleep apnea, chronic liver disease, chronic kidney disease, noncerebrovascular chronic neurologic disease, depression), medications (antiplatelets, statins, other cardiovascular medications), laboratory tests (creatinine, hemoglobin, platelets, white blood cell count, glucose, hemoglobin A1c, estimated glomerular filtration rate, total cholesterol, low-density lipoprotein cholesterol, high-density lipoprotein cholesterol, triglycerides, B-natriuretic peptide, troponin), and imaging (echocardiographic estimation of ejection fraction) (Supplemental Table 3).

## Comparison groups

Three prespecified comparisons were performed to evaluate incident dementia. First, among patients hospitalized with ACS, incident dementia was compared between those who underwent coronary revascularization (PCI or CABG) and those managed medically without revascularization. Second, incident dementia was compared between patients with ACS who underwent coronary revascularization and patients with stable CAD. This stable CAD group consisted of patients who were not admitted for ACS and who did not undergo a coronary revascularization procedure during the study period. Third, within the revascularized ACS cohort, incident dementia was compared between patients undergoing PCI and those undergoing CABG.

### Statistical analysis

Descriptive statistics. Bivariate analyses were performed to compare covariates between revascularized ACS patients vs medically managed ACS patients. The covariates were expressed as frequencies (proportions) or mean ± SD and median (IQR), as appropriate. Comparisons involving categorical variables were performed using the Chi-square or Fisher exact test. Continuous variables were compared using the Student’s *t* test. Two-sided *P* values of <0.05 were considered statistically significant.

Survival analysis. Patients were followed up from 1 year after index hospitalization until the first occurrence of dementia, death, health-plan disenrollment, or study end (December 31, 2024). Patients censored within the 1-year period after the index hospitalization were not included in the survival analyses. Kaplan-Meier curves were utilized to demonstrate the proportion free of dementia stratified by coronary revascularization status. Comparisons between revascularized vs medically managed ACS patients were made using log-rank test. Annual crude incident rates were calculated by dividing the number of new dementia diagnoses by the total time at risk and graphed with a locally weighted scatter plot smoothing (LOWESS) curve. Fine-Gray proportional sub-distribution hazards models, which account for the competing risk of death, were used to estimate sub-HRs, 95% CI, and associated *P* values in 2 ways: 1) full cohort with no adjustment; and 2) propensity-matched cohort (with residual covariate adjustment if necessary). The original power analysis is shown in Supplemental Table 4. All statistical analyses were performed using SAS software version 9.4 (SAS). Kaplan-Meier curves–associated log-rank tests and cumulative incidence curves were done in R using the *survival* and *survminer* packages.

### Propensity-matched cohorts

For each of the comparison groups mentioned earlier, propensity-matched sub-cohorts were created. Logistic regression was used to calculate propensity scores based on baseline demographic and clinical characteristics. For the secondary and tertiary comparison groups, propensity scores were calculated using all patient covariates. For the primary comparison (patients with medically managed ACS), due to cohort size constraints, match balance was only achievable on a subset of core covariates: sex, race/ethnicity, neighborhood deprivation index, BMI, smoking status, and Elixhauser comorbidity index. For this comparison only, multivariable cox regression, including the additional covariates not used to calculate the propensity scores, was used on the propensity-matched dataset. In the case of the primary comparison, one medically managed ACS patient was selected for each revascularized patient. In the case of the secondary comparison, up to three stable CAD patients were selected for each revascularized patient. In the case of the tertiary comparison, up to three PCI patients were selected for each CABG patient. For all matched datasets, the greedy selection method was used to match control patients to exposure patients. Details on matching and matching diagnostics can be found in the supplementary section (Supplemental Table 5). For analyses of incident dementia, individuals with prevalent dementia at baseline were excluded before derivation of baseline covariates. Therefore, dementia was not included in the calculation of the Elixhauser comorbidity index or propensity score–matching procedures.

### Ethics

The study was approved by the KPNC Institutional Review Board with a waiver of written informed consent in accordance with the Medical Research Involving Human Subjects Act due to no direct patient contact.

## Results

### Overview

At baseline, revascularized patients were younger (74.8 ± 6.9 vs 77.1 ± 8.2 years; *P* < 0.001), were more often male (66.2% vs 54.0%; *P* < 0.001), and had lower Elixhauser multimorbidity scores (4.6 ± 2.8 vs 5.4 ± 3.0; *P* < 0.001). Distribution of race and ethnicity differed between the revascularized and medically managed ACS patients with a smaller proportion of White patients in the revascularized group (62.9% vs 64.7%). Revascularized patients had significantly lower baseline prevalence of individual conditions such as hypertension (83.5% vs 88.3%), diabetes (42.0% vs 44.4%), cerebrovascular diseases (16.2% vs 22.3%), noncerebrovascular chronic neurological disease (8.5% vs 12.5%), depression (12.7% vs 15.2%), as well as lower medication use (antiplatelets 12.0% vs 14.5%, statins 55.7% vs 59.7%, other cardiovascular medications 73.2% vs 77.0%). Revascularized patients also had a higher troponin value (Table 1).

**Table 1:** Baseline Demographics and Clinical Characteristics in Patients Hospitalized With Acute Coronary Syndrome Stratified by Revascularization Strategy^a^

	Total (N = 25,176)	Coronary Revascularization (n = 8,840)	No Coronary Revascularization (n = 16,336)	*P* Value
Age at study entry, y				<0.001
Mean ± SD	76.3 ± 7.8	74.8 ± 6.9	77.1 ± 8.2	
Min-Max	65.0-103.0	65.0-101.0	65.0-103.0	
Median (IQR)	75.0 (70.0-82.0)	74.0 (69.0-80.0)	76.0 (70.0-83.0)	
Sex				<0.001
Female	10,500 (41.7)	2,983 (33.7)	7,517 (46.0)	
Male	14,675 (58.3)	5,856 (66.2)	8,819 (54.0)	
Race and ethnicity				<0.001
Asian/Pacific Islander	2,906 (11.5)	1,233 (13.9)	1,673 (10.2)	
Black or African American	1,610 (6.4)	448 (5.1)	1,162 (7.1)	
Hispanic/Latino	2,924 (11.6)	1,017 (11.5)	1,907 (11.7)	
More than one race	1,500 (6.0)	546 (6.2)	954 (5.8)	
Other/Unknown	101 (0.4)	35 (0.4)	66 (0.4)	
White	16,135 (64.1)	5,561 (62.9)	10,574 (64.7)	
Neighborhood Deprivation Index Quintiles				<0.001
1 (least deprived)	2,921 (11.6)	1,366 (15.5)	1,555 (9.5)	
2	5,189 (20.6)	2,016 (22.8)	3,173 (19.4)	
3	5,661 (22.5)	1,961 (22.2)	3,700 (22.6)	
4	4,146 (16.5)	1,260 (14.3)	2,886 (17.7)	
5 (most deprived)	3,166 (12.6)	833 (9.4)	2,333 (14.3)	
Body mass index, kg/m^2^				<0.001
<25	7,345 (29.2)	2,330 (26.4)	5,015 (30.7)	
25-29.9	8,978 (35.7)	3,462 (39.2)	5,516 (33.8)	
30-39.9	6,847 (27.2)	2,506 (28.3)	4,341 (26.6)	
>40	1,037 (4.1)	343 (3.9)	694 (4.2)	
Missing	969 (3.8)	199 (2.3)	770 (4.7)	
Elixhauser comorbidity index				<0.001
Mean ± SD	5.1 ± 3.0	4.6 ± 2.8	5.4 ± 3.0	
Min-Max	0.0-19.0	0.0-17.0	0.0-19.0	
Median (IQR)	5.0 (3.0-7.0)	4.0 (2.0-6.0)	5.0 (3.0-7.0)	
Troponin				<0.001
Mean ± SD	6.2 ± 15.3	9.0 ± 19.9	4.5 ± 11.2	
Min-Max	0.0-102.0	0.0-102.0	0.0-100.0	
Median (IQR)	1.0 (0.2-4.3)	1.2 (0.2-6.2)	0.8 (0.2-3.6)	

a See full table in Supplemental Appendix.

### Unadjusted incident mortality

The median duration of follow-up was 4.8 years (IQR: 2.7-8.1 years). Over the study follow-up, 48.3% of the overall cohort (ACS and stable CAD combined) died (86,767 of 179,475 participants). Among patients with ACS, mortality differed slightly by revascularization status: 41.9% of patients undergoing coronary revascularization died (3,705 of 8,840), including 42.1% of those treated with PCI (3,384 of 8,043) and 40.3% of those treated with CABG (321 of 797). Mortality among patients diagnosed with ACS and those who did not undergo coronary revascularization was 56.3% (9,201 of 16,336). Among patients with stable CAD, 47.9% died during follow-up (73,861 of 154,299).

### Unadjusted incident dementia

**Figure 1:**
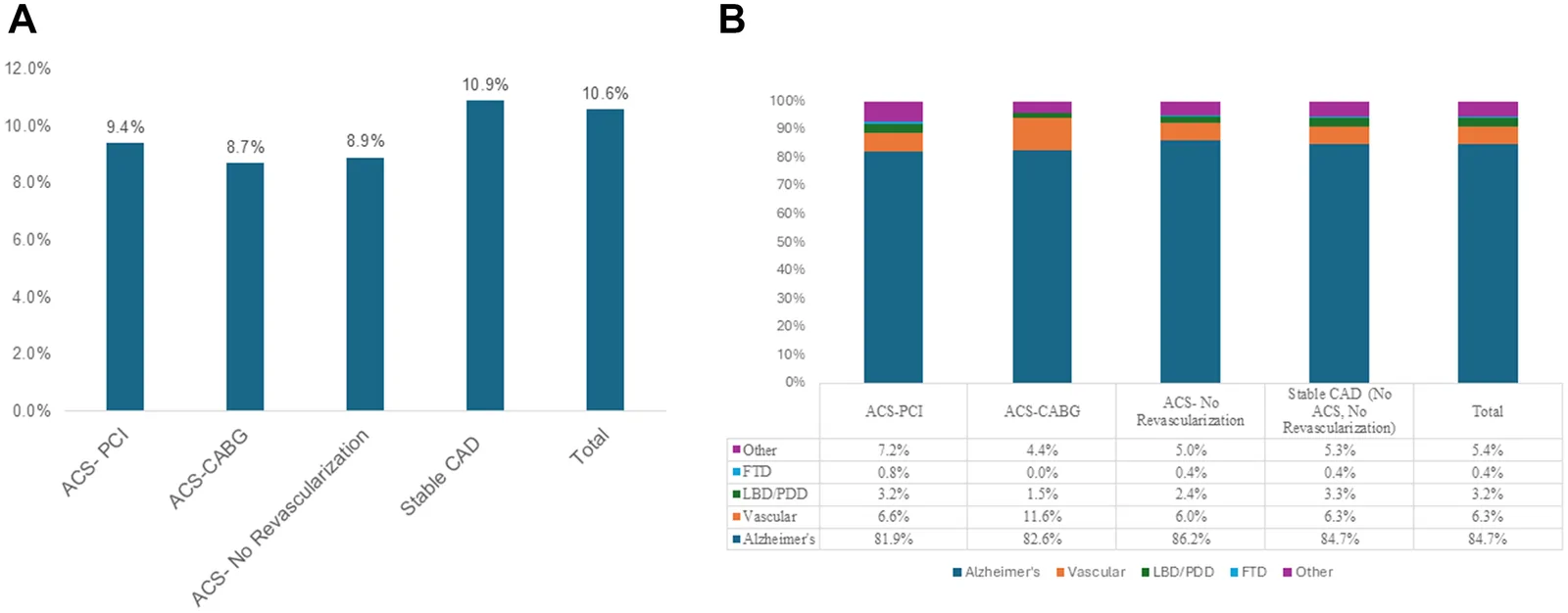
Cumulative Incidence of Dementia Cumulative incidence of incident dementia after a 1-year washout period among older adults with acute coronary syndrome (ACS) and stable coronary artery disease (CAD). (A) All-cause dementia. (B) Dementia subtypes. CABG = coronary artery bypass grafting; PCI = percutaneous coronary intervention.

Over the study follow-up, 9.0% (2,272 of 25,176) of patients with an ACS developed dementia: (total coronary revascularization: 9.3% [823 of 8,840], PCI: 9.4% [754 of 8,043], CABG: 8.7% [69 of 797], no coronary revascularization: 8.9% [14,491 of 16,336]). Among those with stable CAD, 10.9% (16,832 of 154,299) developed dementia over the study period. Figure 1 shows the differences in overall dementia incidence (panel A) and dementia subtypes (panel B) between patients who underwent coronary revascularization and those who did not. Among the dementia subtypes, Alzheimer disease was the most common (84.8%), followed by vascular dementia (6.3%), and Lewy body or Parkinson disease dementia (3.1%).

**Figure 2:**
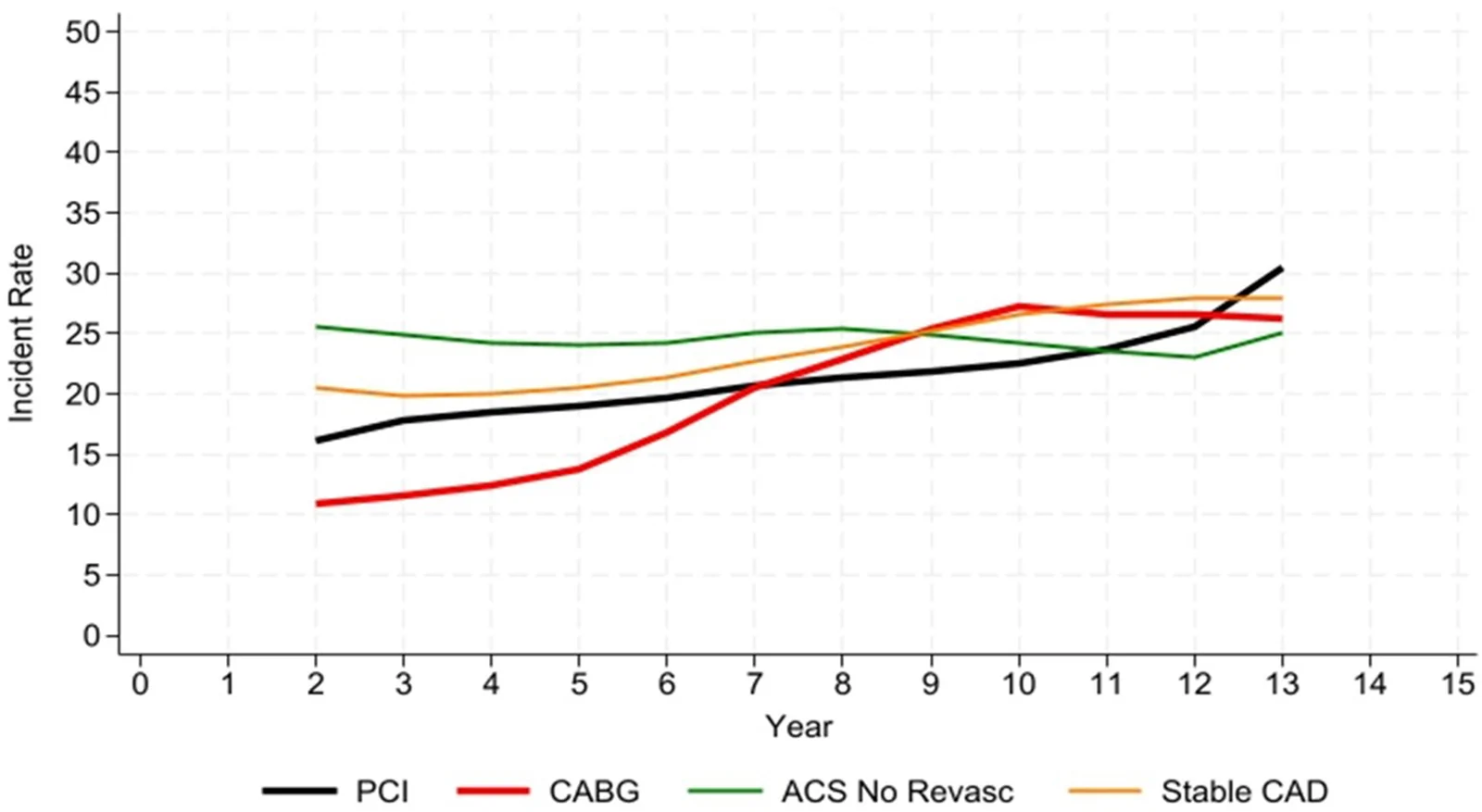
Unadjusted Temporal Trends in Dementia Incidence Unadjusted incidence rates of dementia (per 1,000 person-years) following a 1-year washout among older adults stratified by coronary artery disease status and revascularization strategy. Annual incident dementia rates per 1,000 person-years over follow-up are displayed using locally weighted scatterplot smoothing (LOWESS) curves for percutaneous coronary intervention (PCI) (black line), coronary artery bypass grafting (CABG) (red line), acute coronary syndrome without revascularization (green line), and stable coronary artery disease (orange line). CABG and PCI groups show lower early rates with gradual increases over time, whereas ACS without revascularization begins with the highest rates that remain essentially stable. Stable CAD demonstrates intermediate but slowly increasing rates. Over time, incidence curves converge, with minimal variance between groups by later study years. ACS = acute coronary syndrome; CAD = coronary artery disease.

Annual crude dementia incidence rates per 1,000 person-years demonstrated that CABG and PCI groups had lower early rates that gradually increased over time, whereas medically managed ACS began with the highest rates and remained elevated. Patients with stable CAD demonstrated intermediate but slowly increasing rates, with group differences narrowing in later years (Figure 2).

The unadjusted sub-hazard ratio, accounting for competing risk, was 0.88 (95% CI: 0.80-0.95, *P* = 0.002) when compared with the ACS group without revascularization and 0.71 (95% CI: 0.70 to 0.73, *P* < 0.0001) when compared with stable CAD. The unadjusted sub-HR for CABG vs PCI for incident dementia was 0.87 (95% CI: 0.68-1.11; *P* = 0.25) (Table 2).

**Table 2:** Competing Risk Analysis After 1-Year Washout of Incident Dementia in Older Adults

Analysis Cohort	Sub-HR	95% CI	*P* Value
ACS with coronary revascularization vs ACS without revascularization			
Full cohort (n = 20,770)			
Unadjusted	0.88	(0.80-0.95)	0.002
PS matched cohort (n = 15,231)			
Adjusted via matching and residual covariates	1.05	(0.95-1.17)	0.36
ACS with coronary revascularization vs Stable CAD			
Full cohort (n = 143,372)			
Unadjusted	0.71	(0.70-0.73)	<0.0001
PS matched cohort (n = 29,804)			
Adjusted via matching	0.93	(0.86-1.01)	0.073
CABG vs PCI			
Full cohort (n = 7,981)			
Unadjusted	0.87	(0.68-1.11)	0.25
PS matched cohort (n = 2,781)			
Adjusted via matching	0.98	(0.75-1.28)	0.88

ACS = acute coronary syndrome; CABG = coronary artery bypass grafting; CAD = coronary artery disease; PCI = percutaneous coronary intervention; PS = propensity score.

### Propensity score–matched incident dementia risk

In the propensity score–matched cohort comparing ACS with coronary revascularization vs ACS without revascularization, the adjusted estimate was 1.05 (95% CI: 0.95-1.17; *P* = 0.36). When compared with stable CAD, the adjusted sub-hazard ratio in the PS-matched analysis was 0.93 (95% CI: 0.86-1.01; *P* = 0.073). For CABG vs PCI, adjusted analyses yielded a sub-hazard ratio of 0.98 (95% CI: 0.75-1.28; *P* = 0.88) in the PS-matched cohort (Table 2, Central Illustration).

**Central Illustration:**
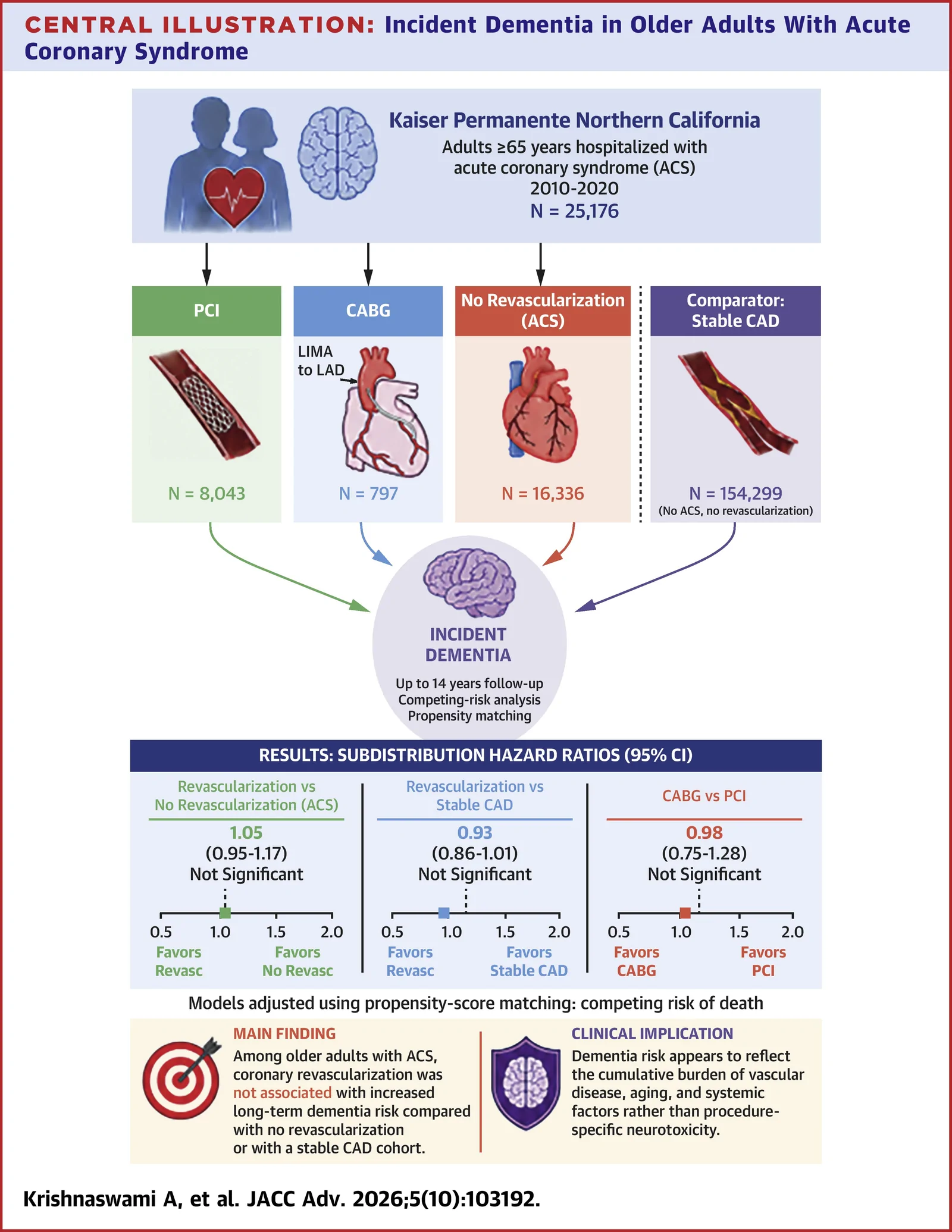
Incident Dementia in Older Adults With Acute Coronary Syndrome CABG = coronary artery bypass grafting; CAD = coronary artery disease; PCI = percutaneous coronary intervention.

**Figure 3:**
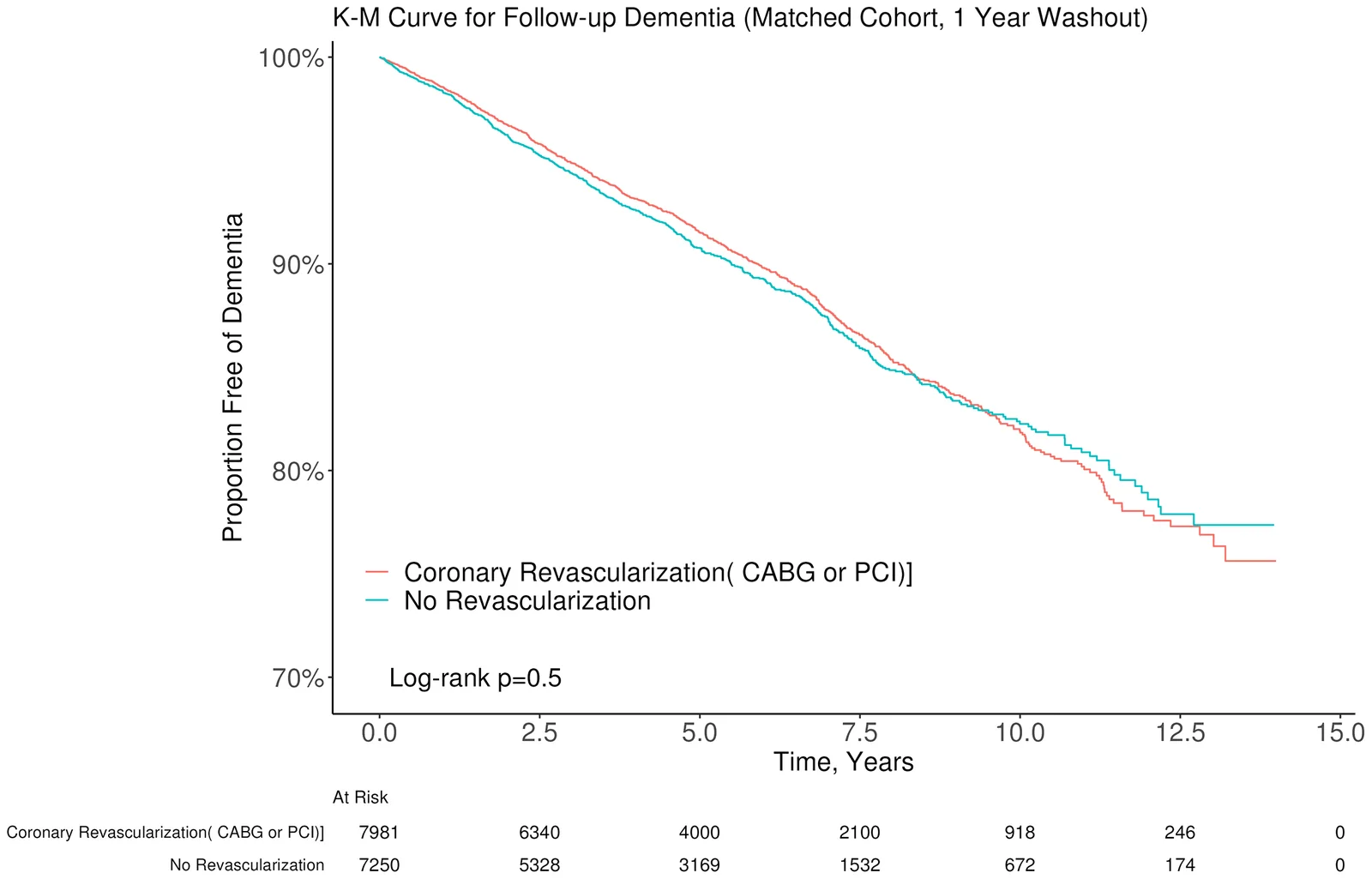
Dementia-Free Survival in Matched ACS Cohorts Unadjusted Kaplan-Meier curves of dementia-free survival after a 1-year washout in propensity score–matched patients with ACS undergoing coronary revascularization compared with those managed without coronary revascularization. CABG = coronary artery bypass grafting; K-M = Kaplan-Meier; PCI = percutaneous coronary intervention.

**Figure 4:**
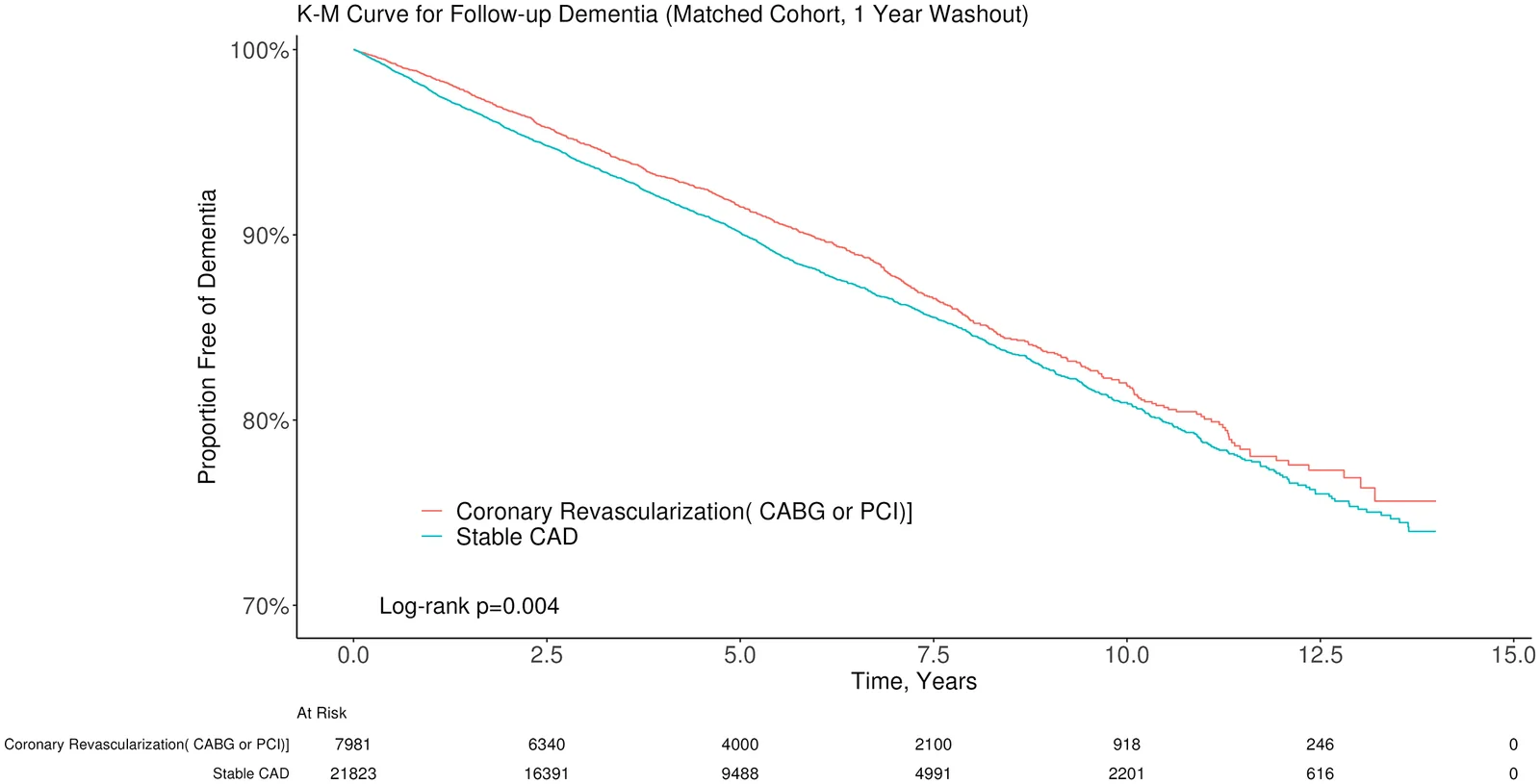
Dementia-Free Survival: Revascularized ACS vs Stable CAD Unadjusted Kaplan-Meier curves demonstrating the proportion of patients remaining free of incident dementia after a 1-year washout in propensity score–matched cohorts comparing patients with acute coronary syndrome (ACS) undergoing coronary revascularization with patients with stable coronary artery disease (CAD) without ACS or coronary revascularization. CABG = coronary artery bypass grafting; PCI = percutaneous coronary intervention.

The accompanying Kaplan-Meier curves demonstrated no statistically significant difference between ACS patients who underwent coronary revascularization and those who did not (log rank *P* = 0.50) (Figure 3). The Kaplan-Meier curves in those with ACS who underwent coronary revascularization compared with the curves in those with stable CAD were noted to be significant (log rank *P* = 0.004) (Figure 4). The Kaplan-Meier curve between coronary revascularization strategies (CABG vs PCI) again demonstrated to be nonsignificant (*P* = 0.40) (Supplemental Figure 1). Supplemental Figure 2 shows a sensitivity analysis Kaplan-Meier curve for incident dementia comparing revascularized and nonrevascularized patients with ACS, without a washout period.

## Discussion

Distinct from previous reports, this large, community-based study with long-term follow-up of older adults hospitalized with ACS directly compared revascularized and nonrevascularized patients with ACS along with a second comparator (patients with stable CAD) and applied propensity-matched modeling with competing-risk methods to provide a more rigorous assessment of dementia risk. Within this carefully characterized cohort followed up to 14 years, dementia affected approximately 1 in 12 patients (8.2%) during follow-up. The predominant clinical subtype was Alzheimer’s (84.8%), followed by vascular disease (6.3%), and Lewy body/Parkinson disease dementia (3.1%). Despite baseline heterogeneity in unadjusted dementia incidence across the four groups, long-term incidence rates demonstrated convergence, approaching approximately 25 to 30 cases per 1,000 person-years after 10 years of follow-up. After adjustment via propensity matching, no statistically significant differences in the risk of incident dementia were observed across groups: The risk was similar for patients with ACS irrespective of whether they underwent revascularization, and not significantly different when patients with ACS with revascularization were compared with those with stable CAD, and comparable between CABG and PCI. Collectively, these findings suggest that incident dementia risk was not significantly associated with coronary revascularization status or revascularization strategy after adjustment for measured confounders and the competing risk of death, and that underlying patient characteristics and vascular risk factors may play an important role in dementia development across the spectrum of coronary disease.

Our findings align with those of Whitlock et al^5^ who examined community-dwelling older adults in the Health and Retirement Study and found no significant difference in the rate of memory decline and probability of dementia between patients who underwent CABG and those who underwent PCI. Their work demonstrated that the type of coronary revascularization was not an independent risk factor for late-life cognitive trajectory. Our results extend these observations to a substantially larger, healthcare system-based cohort of older adults hospitalized with ACS, incorporating both revascularized, medically managed patients along with a stable CAD second comparator while accounting for competing mortality risk. The absence in our study of statistically significant differences in dementia risk across revascularization strategies likely reflects the potential dominant influence of chronic vascular and systemic factors over transient procedural effects while not excluding it.

Our results are also consistent with findings of a comprehensive systematic review by the Agency of Healthcare Research and Quality, one of the largest studies in this area, and other systematic reviews on cognitive outcomes after cardiovascular procedures,^3^^,^^4^ which found no consistent association between cardiac surgery or PCI and long-term cognitive decline. Our study extends prior work by including two clinically relevant comparator groups, patients with ACS managed without revascularization and patients with stable CAD, and by applying competing-risk methods. Although the Agency of Healthcare Research and Quality study noted transient postoperative changes, long-term impairment was uncommon after accounting for age, vascular burden, and comorbidities. These findings support the view that late-life cognitive decline after ACS probably reflects underlying systemic and vascular factors rather than procedure-specific neurotoxicity.

Beyond procedural and selection factors, our findings likely reflect the strong contribution of shared and potentially modifiable risk factors to late-life cognitive decline. The Lancet Commission on Dementia Prevention, Intervention, and Care^13^ estimated that up to 45% of dementia cases may be modifiable by addressing risk factors of early-, mid-, and late-life exposure morbidities. These include education, hypertension, diabetes, obesity, smoking, depression, social isolation, physical inactivity, and hearing loss. These health conditions overlap substantially with the risk profile of patients with CAD. Vascular and lifestyle factors such as hypertension, diabetes, and inactivity contribute to chronic inflammation and small-vessel brain injury.^13^ The incidence of dementia after ACS or revascularization likely reflects this cumulative burden. Therefore, managing cardiovascular risk factors remains central not only to improving heart outcomes but also to preserving cognitive health in later life.

Our results along with the current body of current evidence underscore the need for an integrated approach to cardiovascular and cognitive health in older adults. As highlighted by both the American Heart Association Scientific Statement on ACS in older populations^7^ and the Lancet Commission,^13^ prevention of dementia begins with aggressive management of vascular and behavioral risk factors across the life course. In the setting of a learning health system, linking cardiac and geriatric care pathways could enable the systematic identification of patients at high risk of both recurrent cardiovascular events and cognitive decline. Routine assessment of cognitive and functional status, optimization of secondary prevention therapies, and incorporation of multidomain interventions such as addressing physical activity, blood pressure control, social engagement, and sensory health (vision, hearing) represent opportunities to extend the benefits of cardiovascular care into the domain of cognitive resilience. Such alignment of cardiology, geriatrics, and preventive medicine may help reframe revascularization decisions not only in terms of survival and symptom relief but also in their potential to preserve independence and quality of life.

### Study Limitations

This study has several limitations. First, dementia diagnoses were based on *ICD* codes rather than standardized neuropsychological testing, which might have led to a potential under ascertainment of milder cognitive impairment. Second, despite the rigorous statistical adjustment using propensity score matching, residual confounding from unmeasured factors such as baseline cognitive reserve, frailty, or social determinants of health cannot be excluded. Third, we lacked direct measures of procedural characteristics, such as pump time, which may have an influence on cognitive outcomes. We also did not gather data to demonstrate a stroke-mediated increase in dementia. Fourth, some of the medically managed patients diagnosed with ACS may have had other diagnoses such as pericarditis and myocarditis that may be similar to an ACS presentation. Moreover, our results reflect practice patterns within an integrated healthcare delivery system and may not generalize to other populations or healthcare settings. Lastly, we would like to reiterate that our findings are observational in nature and should be interpreted as associations rather than causal effects.

Despite these limitations, this study has notable strengths, including a large and diverse insured population in an integrated health system, which enabled the comprehensive capture of diagnoses, procedures, and outcomes over up to 14 years of follow-up. By incorporating both nonrevascularized ACS and stable CAD comparators, it provides one of the most complete assessments to date of dementia risk across the full spectrum of coronary disease.

## Conclusions

In this large, community-based cohort of older adults with ACS, no significant association was observed between coronary revascularization and long-term risk of incident dementia. These findings suggest that factors such as overall vascular burden, cardiometabolic health, and patient characteristics may play a greater role in determining long-term dementia risk than coronary revascularization strategy. Therefore, efforts to preserve cognitive health should prioritize optimization of cardiovascular risk factors, early recognition of cognitive vulnerability, and integration of geriatric principles into cardiovascular care.

Perspectives **COMPETENCY IN MEDICAL KNOWLEDGE:** Among older adults with acute coronary syndrome, coronary revascularization with PCI or CABG was not associated with an increased long-term risk of incident dementia compared with no revascularization or stable coronary artery disease. Concerns regarding dementia risk alone should not preclude consideration of clinically indicated coronary revascularization.

**COMPETENCY IN PATIENT CARE:** Long-term dementia risk following acute coronary syndrome appears more closely related to the cumulative burden of vascular disease, aging, and multimorbidity than to procedure-specific neurotoxicity. Clinicians should prioritize optimization of vascular risk factors and comprehensive longitudinal care to support cognitive health in older adults with coronary artery disease.

**TRANSLATIONAL OUTLOOK:** Future research incorporating serial cognitive assessments, neuroimaging, and biomarkers is needed to further clarify cognitive trajectories following ACS and stable CAD to identify potentially modifiable pathways for dementia prevention in older adults with coronary artery disease.

### Declaration of Generative AI and AI-Assisted Technologies in the Writing Process

Artificial intelligence-based language assistance was used for grammatical editing and to reduce word count. The AI tool did not analyze data, interpret results, or influence the scientific conclusions. All final text were reviewed, edited, and approved by the authors. The AI tool was used to create the central illustration with numerous iterations to obtain an acceptable image.

## Funding support and author disclosures

This study received Kaiser Permanente Community Benefit Grant (CH6259). Dr Bhatt discloses the following relationships: is part of the advisory board at Angiowave, Antlia Bioscience, Bayer, Boehringer Ingelheim, CellProthera, Cereno Scientific, E-Star Biotech, High Enroll, Janssen, Level Ex, McKinsey, Medscape Cardiology, Merck, NirvaMed, Novo Nordisk, Repair Biotechnologies, Stasys, SandboxAQ (stock options), Tourmaline Bio, and Viatris; is part of the board of directors at American Heart Association New York City, Angiowave (stock options), Bristol Myers Squibb (stock), DRS.LINQ (stock options), and High Enroll (stock); is a consultant for Alnylam, Altimmune, Broadview Ventures, Corcept Therapeutics, Corsera, GlaxoSmithKline, Hims, SERB, SFJ, Summa Therapeutics, and Worldwide Clinical Trials; is part of the data monitoring committees at Acesion Pharma, Assistance Publique-Hôpitaux de Paris, Baim Institute for Clinical Research, Boston Scientific (Chair, PEITHO trial), Cleveland Clinic, Contego Medical (Chair, PERFORMANCE 2), Duke Clinical Research Institute, Mayo Clinic, Mount Sinai School of Medicine (for the ABILITY-DM trial, funded by Concept Medical; for ALLAY-HF, funded by Alleviant Medical), Novartis, and Population Health Research Institute; Rutgers University (for the NIH-funded MINT Trial); receives honoraria from the American College of Cardiology (Senior Associate Editor, Clinical Trials and News, ACC.org; Chair, ACC Accreditation Oversight Committee), Arnold and Porter law firm (work related to Sanofi/Bristol-Myers Squibb clopidogrel litigation), Baim Institute for Clinical Research (AEGIS-II executive committee funded by CSL Behring), Belvoir Publications (Editor in Chief, Harvard Heart Letter), Canadian Medical and Surgical Knowledge Translation Research Group (clinical trial steering committees), CSL Behring (AHA lecture), Duke Clinical Research Institute, Engage Health Media, HMP Global (Editor in Chief, *Journal of Invasive Cardiology*), Medtelligence/ReachMD (CME steering committees), MJH Life Sciences, Oakstone CME (Course Director, Comprehensive Review of Interventional Cardiology), Philips (Becker's Webinar on AI), Population Health Research Institute, WebMD (CME steering committees), Wiley (steering committee); discloses other conflicts such as Clinical Cardiology (Deputy Editor, unpaid); Progress in Cardiovascular Diseases (Deputy Editor, unpaid); Added Health (Editorial Board; stock options); patent with Sotagliflozin (named on a patent for sotagliflozin assigned to Brigham and Women's Hospital who assigned to Lexicon; neither I nor Brigham and Women's Hospital receive any income from this patent); receives research funding from Abbott, Acesion Pharma, Afimmune, Alnylam, Amarin, Amgen, AstraZeneca, Atricure, Bayer, Boehringer Ingelheim, Boston Scientific, CellProthera, Cereno Scientific, Chiesi, Cleerly, CSL Behring, Faraday Pharmaceuticals, Fractyl, Idorsia, Janssen, Javelin, Lexicon, Lilly, Medtronic, Merck, MiRUS, Moderna, Novartis, Novo Nordisk, Pfizer, PhaseBio, Regeneron, Reid Hoffman Foundation, Roche, Sanofi, Stasys, and 89Bio; receives royalties from Elsevier (Editor, Braunwald’s Heart Disease); and is a site co-investigator at Cleerly. Dr Aggarwal’s and Dr Mielke’s efforts were supported by U24 AG082930. Dr Mielke has also served on scientific advisory boards and/or has consulted for Biogen, Eisai, LabCorp, Lilly, Merck, and Roche Siemens Healthier; received speaking honorariums from Novo Nordisk, PeerView Institute, and Roche; and receives grant support from the National Institute of Health, Department of Defense, Alzheimer’s Association, and Davos Alzheimer’s Collaborative. Dr Volgman receives research funding from Novartis, Janssen, and the NIH. She is a national lead investigator for the Librexia study by Janssen. She is on a data safety monitoring board of Chiesi Pharmaceuticals and on the advisory board of Zoll Pharmaceuticals. She is a consultant for Regeneron and Sanofi. Dr Maurer has research or grant support from Alexion Pharmaceuticals, Attralus Therapeutics, BridgeBio, Intellia Therapeutics, and Ionis Pharmaceuticals and also receives consulting fees from Alnylam Pharmaceuticals, AstraZeneca, Intellia Therapeutics, Ionis Pharmaceuticals, Bayer, Novo Nordisk, and Roche. Dr Nanna reports current research support from the 10.13039/100005485American College of Cardiology Foundation, the 10.13039/100006093Patient-Centered Outcomes Research Institute (PCORI), the Yale Claude D. Pepper Older Americans Independence Center (P30AG021342), and the 10.13039/100000049National Institute on Aging (K76AG088428) and personal fees from HeartFlow, Inc, Merck, and Novo Nordisk. Dr Damluji receives research funding from the Pepper Scholars Program of the Johns Hopkins University Claude D. Pepper Older Americans Independence Center (OAIC) funded by the 10.13039/100000049NIA P30-AG021334; and receives mentored patient-oriented research career development award from the 10.13039/100000050National Heart, Lung, and Blood Institute K23-HL153771-01. All other authors have reported that they have no relationships relevant to the contents of this paper to disclose.
