# Supplementary material for: Incidence of Dementia in Older Adults Hospitalized With Acute Coronary Syndrome: Insights From KP-Brain-Heart Health Study
Source: JACC Adv. 2026 Aug 28;5(10):103192. doi: 10.1016/j.jacadv.2026.103192 (PMC13545510; doi:10.1016/j.jacadv.2026.103192)

Supplementary Section

Supplementary Table 1. Internal Diagnosis codes used to identify Acute Coronary Syndrome/Stable CAD.

Note: Kaiser Permanente codes diagnoses using an internal coding system that maps to ICD9/10 codes. The below table shows the internal codes ("dx_id") that were used to identify ACS and Stable CAD as inclusionary conditions.

Internal DX_ID	Internal Diagnosis Description	ICD9 Code	ICD10 Code	ACS/Stable CAD
190121783	MI, ACUTE, LATERAL WALL, INITIAL EPISODE OF CARE	410.51		**ACS**
121208764	ACUTE ST ELEVATION MI, SEPTUM, SUBSEQ EPISODE OF CARE.	410.82	I21.29	**ACS**
121208763	ACUTE ST ELEVATION MI, RIGHT CORONARY ARTERY, SUBSEQ EPISODE OF CARE.	410.82	I21.11	**ACS**
121208762	ACUTE ST ELEVATION MI, POSTEROLATERAL WALL, SUBSEQ EPISODE OF CARE.	410.52	I21.29	**ACS**
121208761	ACUTE ST ELEVATION MI, POSTEROBASAL WALL, SUBSEQ EPISODE OF CARE.	410.62	I21.29	**ACS**
121208760	ACUTE ST ELEVATION MI, POSTERIOR WALL, SUBSEQ EPISODE OF CARE.	410.62	I21.29	**ACS**
121208759	ACUTE ST ELEVATION MI, LEFT MAIN CORONARY ARTERY, SUBSEQ EPISODE OF CARE.	410.12	I21.01	**ACS**
121208758	ACUTE ST ELEVATION MI, LEFT CORONARY ARTERY, SUBSEQ EPISODE OF CARE.	410.82	I21.29	**ACS**
121208757	ACUTE ST ELEVATION MI, LEFT CIRCUMFLEX CORONARY ARTERY, SUBSEQ EPISODE OF CARE.	410.82	I21.21	**ACS**
121208756	ACUTE ST ELEVATION MI, LEFT ANTERIOR DESCENDING CORONARY ARTERY, SUBSEQ EPISODE OF CARE.	410.12	I21.02	**ACS**
121208755	ACUTE ST ELEVATION MI, LATERAL WALL, SUBSEQ EPISODE OF CARE.	410.52	I21.29	**ACS**
121208754	ACUTE ST ELEVATION MI, INFEROPOSTERIOR WALL, SUBSEQ EPISODE OF CARE.	410.32	I21.11	**ACS**
121208753	ACUTE ST ELEVATION MI, INFEROLATERAL WALL, SUBSEQ EPISODE OF CARE.	410.22	I21.19	**ACS**
121208752	ACUTE ST ELEVATION MI, INFERIOR WALL, SUBSEQ EPISODE OF CARE.	410.42	I21.19	**ACS**
121208751	ACUTE ST ELEVATION MI, ATRIUM, SUBSEQ EPISODE OF CARE.	410.82	I21.29	**ACS**
121208750	ACUTE ST ELEVATION MI, ANTEROSEPTAL, SUBSEQ EPISODE OF CARE.	410.12	I21.09	**ACS**
121208749	ACUTE ST ELEVATION MI, ANTEROLATERAL WALL, SUBSEQ EPISODE OF CARE.	410.02	I21.09	**ACS**

Internal DX_ID	Internal Diagnosis Description	ICD9 Code	ICD10 Code	ACS/Stable CAD
121208748	ACUTE ST ELEVATION MI, ANTEROAPICAL, SUBSEQ EPISODE OF CARE.	410.12	I21.09	**ACS**
121208747	ACUTE ST ELEVATION MI, ANTERIOR WALL, SUBSEQ EPISODE OF CARE.	410.12	I21.09	**ACS**
121208746	ACUTE ST ELEVATION MI, SUBSEQ EPISODE OF CARE.	410.92	I21.3	**ACS**
121208745	ACUTE NON ST ELEVATION MI, SUBSEQ EPISODE OF CARE.	410.72	I21.4	**ACS**
121208744	ACUTE ST ELEVATION MI, SEPTUM, INITIAL EPISODE OF CARE.	410.81	I21.29	**ACS**
121208743	ACUTE ST ELEVATION MI, RIGHT CORONARY ARTERY, INITIAL EPISODE OF CARE.	410.81	I21.11	**ACS**
121208742	ACUTE ST ELEVATION MI, POSTEROLATERAL WALL, INITIAL EPISODE OF CARE.	410.51	I21.29	**ACS**
121208741	ACUTE ST ELEVATION MI, POSTEROBASAL WALL, INITIAL EPISODE OF CARE.	410.61	I21.29	**ACS**
121208740	ACUTE ST ELEVATION MI, POSTERIOR WALL, INITIAL EPISODE OF CARE.	410.61	I21.29	**ACS**
121208739	ACUTE ST ELEVATION MI, LEFT MAIN CORONARY ARTERY, INITIAL EPISODE OF CARE.	410.11	I21.01	**ACS**
121208738	ACUTE ST ELEVATION MI, LEFT CORONARY ARTERY, INITIAL EPISODE OF CARE.	410.81	I21.29	**ACS**
121208737	ACUTE ST ELEVATION MI, LEFT CIRCUMFLEX CORONARY ARTERY, INITIAL EPISODE OF CARE.	410.81	I21.21	**ACS**
121208736	ACUTE ST ELEVATION MI, LEFT ANTERIOR DESCENDING CORONARY ARTERY, INITIAL EPISODE OF CARE.	410.11	I21.02	**ACS**
121208735	ACUTE ST ELEVATION MI, LATERAL WALL, INITIAL EPISODE OF CARE.	410.51	I21.29	**ACS**
121208734	ACUTE ST ELEVATION MI, INFEROPOSTERIOR WALL, INITIAL EPISODE OF CARE.	410.31	I21.11	**ACS**
121208733	ACUTE ST ELEVATION MI, INFEROLATERAL WALL, INITIAL EPISODE OF CARE.	410.21	I21.19	**ACS**
121208732	ACUTE ST ELEVATION MI, INFERIOR WALL, INITIAL EPISODE OF CARE.	410.41	I21.19	**ACS**
121208731	ACUTE ST ELEVATION MI, ATRIUM, INITIAL EPISODE OF CARE.	410.81	I21.29	**ACS**
121208730	ACUTE ST ELEVATION MI, ANTEROSEPTAL, INITIAL EPISODE OF CARE.	410.11	I21.09	**ACS**
121208729	ACUTE ST ELEVATION MI, ANTEROLATERAL WALL, INITIAL EPISODE OF CARE.	410.01	I21.09	**ACS**
121208728	ACUTE ST ELEVATION MI, ANTEROAPICAL, INITIAL EPISODE OF CARE.	410.11	I21.09	**ACS**

Internal DX_ID	Internal Diagnosis Description	ICD9 Code	ICD10 Code	ACS/Stable CAD
121208727	ACUTE ST ELEVATION MI, ANTERIOR WALL, INITIAL EPISODE OF CARE.	410.11	I21.09	**ACS**
121208726	ACUTE ST ELEVATION MI, INITIAL EPISODE OF CARE.	410.91	I21.3	**ACS**
121208725	ACUTE NON ST ELEVATION MI, INITIAL EPISODE OF CARE.	410.71	I21.4	**ACS**
121141501	ACUTE ST ELEVATION MI, LEFT CIRCUMFLEX CORONARY ARTERY	410.80	I21.21	**ACS**
121141486	CAD W ANGINA AND SPASM	414.00, 413.9	I25.111	**Stable CAD**
121141460	CAD OF CABG, UNSPECIFIED, W ANGINA AND SPASM	414.05, 413.9	I25.701	**Stable CAD**
121141459	CAD OF BYPASS GRAFT OF TRANSPLANTED HEART W UNSPECIFIED ANGINA	414.07, 413.9	I25.769	**Stable CAD**
121141458	CAD OF BYPASS GRAFT OF TRANSPLANTED HEART W ANGINA AND SPASM	414.07, 413.9	I25.761	**Stable CAD**
121141456	CAD OF TRANSPLANTED HEART W UNSPECIFIED ANGINA	414.00, 413.9, V42.1	I25.759	**Stable CAD**
121141455	CAD OF TRANSPLANTED HEART W ANGINA AND SPASM	414.00, 413.9, V42.1	I25.751	**Stable CAD**
121141454	CAD OF TRANSPLANTED HEART W UNSTABLE ANGINA	414.00, 411.1, V42.1	I25.750	**Stable CAD**
121141453	CAD OF NONAUTOLOGOUS BIOLOGICAL CABG W UNSPECIFIED ANGINA	414.03, 413.9	I25.739	**Stable CAD**
121141452	CAD OF NONAUTOLOGOUS BIOLOGICAL CABG W ANGINA AND SPASM	414.03, 413.9	I25.731	**Stable CAD**
121141451	CAD OF NONAUTOLOGOUS BIOLOGICAL CABG W UNSTABLE ANGINA	414.03, 411.1	I25.730	**Stable CAD**
121141450	CAD OF AUTOLOGOUS ARTERY CABG W UNSPECIFIED ANGINA	414.04, 413.9	I25.729	**Stable CAD**
121141449	CAD OF AUTOLOGOUS ARTERY CABG W ANGINA AND SPASM	414.04, 413.9	I25.721	**Stable CAD**
121141448	CAD OF AUTOLOGOUS ARTERY CABG W UNSTABLE ANGINA	414.04, 411.1	I25.720	**Stable CAD**
121141447	CAD OF AUTOLOGOUS VEIN CABG W UNSPECIFIED ANGINA	414.02, 413.9	I25.719	**Stable CAD**
121141446	CAD OF AUTOLOGOUS VEIN CABG W ANGINA AND SPASM	414.02, 413.9	I25.711	**Stable CAD**
121141445	CAD OF AUTOLOGOUS VEIN CABG W UNSTABLE ANGINA	414.02, 411.1	I25.710	**Stable CAD**
121141444	CAD OF CABG, UNSPECIFIED, W UNSPECIFIED ANGINA	414.05, 413.9	I25.709	**Stable CAD**

Internal DX_ID	Internal Diagnosis Description	ICD9 Code	ICD10 Code	ACS/Stable CAD
121141443	CAD OF CABG, UNSPECIFIED, W UNSTABLE ANGINA AND SPASM	414.05, 411.1	I25.700, I25.701	**Stable CAD**
121141442	CAD OF CABG, UNSPECIFIED, W UNSTABLE ANGINA	414.05, 411.1	I25.700	**Stable CAD**
121141441	SILENT MYOCARDIAL ISCHEMIA	414.8	I25.6	**Stable CAD**
121141438	CAD W UNSPECIFIED TYPE ANGINA	414.00, 413.9	I25.119	**Stable CAD**
121141437	CAD W UNSTABLE ANGINA AND SPASM	414.00, 411.1	I25.110, I25.111	**Stable CAD**
121141436	CAD W UNSTABLE ANGINA	414.01, 411.1	I25.110	**Stable CAD**
121141434	POSTINFARCTION ANGINA	413.9	I23.7	**ACS**
121141258	ACUTE ST ELEVATION MI, ATRIUM	410.80	I21.29	**ACS**
121141257	ACUTE ST ELEVATION MI, ANTEROAPICAL	410.10	I21.09	**ACS**
121141256	ACUTE ST ELEVATION MI, POSTEROLATERAL WALL	410.50	I21.29	**ACS**
121141255	ACUTE ST ELEVATION MI, POSTEROBASAL WALL	410.60	I21.29	**ACS**
121141254	ACUTE ST ELEVATION MI, SEPTUM	410.80	I21.29	**ACS**
121141253	ACUTE ST ELEVATION MI, LEFT CORONARY ARTERY	410.80	I21.01	**ACS**
121141252	ACUTE ST ELEVATION MI, POSTERIOR WALL	410.60	I21.29	**ACS**
121141251	ACUTE ST ELEVATION MI, INFEROPOSTERIOR WALL	410.30	I21.11	**ACS**
121141250	ACUTE ST ELEVATION MI, ANTEROSEPTAL	410.10	I21.09	**ACS**
121141249	ACUTE ST ELEVATION MI, LATERAL WALL	410.50	I21.29	**ACS**
121141248	ACUTE ST ELEVATION MI, INFEROLATERAL WALL	410.20	I21.19	**ACS**
121141247	ACUTE ST ELEVATION MI, ANTEROLATERAL WALL	410.0	I21.09	**ACS**
121141246	ACUTE ST ELEVATION MI, RIGHT CORONARY ARTERY	410.80	I21.11	**ACS**
121141245	ACUTE ST ELEVATION MI, INFERIOR WALL	410.40	I21.19	**ACS**

Internal DX_ID	Internal Diagnosis Description	ICD9 Code	ICD10 Code	ACS/Stable CAD
121141244	ACUTE ST ELEVATION MI, ANTERIOR WALL	410.10	I21.09	**ACS**
121126709	HX OF OLD MI W STABLE ANGINA	413.9, 412	I20.8, I25.2	**Stable CAD**
121123964	ATHSCL NONAUTOLOGOUS BIOLOGICAL CABG W OTH ANGINA PECTORIS		I25.738	**Stable CAD**
121123963	ATHSCL AUTOLOGOUS ARTERY CABG W ANG PCTRS W DOCUMENTED SPASM		I25.721	**Stable CAD**
121122836	ATHSCL AUTOLOGOUS VEIN CABG W ANG PCTRS W DOCUMENTED SPASM		I25.711	**Stable CAD**
121122273	ATHSCL BYPASS OF COR ART OF TXPLT HEART W OTH ANG PCTRS		I25.768	**Stable CAD**
121122272	ATHSCL NONAUTOLOGOUS BIOLOGICAL CABG W UNSP ANGINA PECTORIS		I25.739	**Stable CAD**
121121704	ATHSCL CABG, UNSP, W ANGINA PECTORIS W DOCUMENTED SPASM		I25.701	**Stable CAD**
121121131	ATHSCL NATIVE COR ART OF TXPLT HEART W ANG PCTRS W SPASM		I25.751	**Stable CAD**
121120524	ATHSCL BYPASS OF COR ART OF TXPLT HEART W UNSP ANG PCTRS		I25.769	**Stable CAD**
121120523	ATHSCL NONAUTOLOGOUS BIOLOGICAL CABG W UNSTABLE ANG PCTRS		I25.730	**Stable CAD**
121119922	ATHSCL AUTOLOGOUS ARTERY CABG W OTH ANGINA PECTORIS		I25.728	**Stable CAD**
121118843	ATHSCL AUTOLOGOUS VEIN CABG W OTH ANGINA PECTORIS		I25.718	**Stable CAD**
121118302	ATHSCL AUTOLOGOUS ARTERY CABG W UNSP ANGINA PECTORIS		I25.729	**Stable CAD**
121117724	ATHEROSCLEROSIS OF CABG, UNSP, W OTH ANGINA PECTORIS		I25.708	**Stable CAD**
121117143	ATHSCL NATIVE COR ART OF TRANSPLANTED HEART W OTH ANG PCTRS		I25.758	**Stable CAD**
121117142	ATHSCL AUTOLOGOUS VEIN CABG W UNSP ANGINA PECTORIS		I25.719	**Stable CAD**
121116568	ATHSCL AUTOLOGOUS ARTERY CABG W UNSTABLE ANGINA PECTORIS		I25.720	**Stable CAD**
121116567	ATHSCL HEART DISEASE OF NATIVE COR ART W ANG PCTRS W SPASM		I25.111	**Stable CAD**
121116050	ATHEROSCLEROSIS OF CABG, UNSP, W UNSP ANGINA PECTORIS		I25.709	**Stable CAD**
121115508	ATHSCL BYPASS OF COR ART OF TRANSPLANTED HEART W/O ANG PCTRS		I25.812	**Stable CAD**

Internal DX_ID	Internal Diagnosis Description	ICD9 Code	ICD10 Code	ACS/Stable CAD
121115507	ATHSCL NATIVE COR ART OF TRANSPLANTED HEART W UNSP ANG PCTRS		I25.759	**Stable CAD**
121115506	ATHSCL AUTOLOGOUS VEIN CABG W UNSTABLE ANGINA PECTORIS		I25.710	**Stable CAD**
121114420	ATHEROSCLEROSIS OF CABG, UNSP, W UNSTABLE ANGINA PECTORIS		I25.700	**Stable CAD**
121113889	STEMI INVOLVING LEFT ANTERIOR DESCENDING CORONARY ARTERY		I21.02	**ACS**
121112821	ATHSCL HEART DISEASE OF NATIVE COR ART W OTH ANG PCTRS		I25.118	**Stable CAD**
121112820	STEMI INVOLVING OTH CORONARY ARTERY OF INFERIOR WALL		I21.19	**ACS**
121112819	STEMI INVOLVING OTH CORONARY ARTERY OF ANTERIOR WALL		I21.09	**ACS**
121111849	ATHSCL HEART DISEASE OF NATIVE CORONARY ARTERY W/O ANG PCTRS		I25.10	**Stable CAD**
121111181	STEMI INVOLVING LEFT MAIN CORONARY ARTERY		I21.01	**ACS**
121108484	ATHEROSCLEROSIS OF CABG W/O ANGINA PECTORIS		I25.810	**Stable CAD**
121108483	STEMI INVOLVING RIGHT CORONARY ARTERY		I21.11	**ACS**
121100384	STEMI INVOLVING OTH SITES		I21.29	**ACS**
19041090	MYOCARD INFARCT- ACUTE	410.90		**ACS**
19041082	AMI NEC- SUBSEQUENT	410.82		**ACS**
19041081	AMI NEC- INITIAL	410.81		**ACS**
19041080	AMI NEC- UNSPECIFIED	410.80		**ACS**
19041062	TRUE POSTERIOR WALL INFARCT- SUBSEQ	410.62		**ACS**
19041061	TRUE POSTERIOR WALL INFARCT- INIT	410.61		**ACS**
19041032	AMI- INFEROPOSTERIOR WALL- SUBSEQ	410.32		**ACS**
19041031	AMI- INFEROPOSTERIOR WALL- INITIAL	410.31		**ACS**
19041022	AMI- INFEROLATERAL WALL- SUBSEQUENT	410.22		**ACS**

Internal DX_ID	Internal Diagnosis Description	ICD9 Code	ICD10 Code	ACS/Stable CAD
19041021	AMI- INFEROLATERAL WALL- INITIAL	410.21		**ACS**
19041002	AMI- ANTEROLATERAL WALL- SUBSEQUENT	410.02		**ACS**
19041001	AMI- ANTEROLATERAL WALL- INITIAL	410.01		**ACS**
12461578	ATHEROSCL BP GRFT COR ART TPLNT HRT W/RFRCTRY AP		I25.762	**Stable CAD**
12461577	ATHEROSCLEROSIS NATIVE CA TPLNT HRT W/RFRCTRY AP		I25.752	**Stable CAD**
12461576	ATHEROSCLEROSIS NONAUTOLOG BIO CABG W/RFRCTRY AP		I25.732	**Stable CAD**
12461575	ATHEROSCLEROSIS AUTOLOG ART CABG W/REFRACTORY AP		I25.722	**Stable CAD**
12461574	ATHEROSCLEROSIS AUTOLOG VN CABG W/REFRACTORY AP		I25.712	**Stable CAD**
12461573	ATHEROSCLEROSIS CABG UNS W/REFRACTORY AP		I25.702	**Stable CAD**
12461572	ASHD NATIVE COR ART W/REFRACTORY ANGINA PECTORIS		I25.112	**Stable CAD**
12461283	CAD W ANGINA, W HX OF MI		I25.119, I25.2	**Stable CAD**
12461282	CAD W ANGINA, W HX OF CABG		I25.119, Z95.1	**Stable CAD**
12461281	CAD W ANGINA, PRESENCE OF STENT		I25.119, Z95.5	**Stable CAD**
12456197	SUBSEQ TYPE 3 MI, WITHIN 4 WKS OF PREVIOUS MI		I21.A9	**ACS**
12456196	TYPE 3 MI		I21.A9	**ACS**
12456195	SUBSEQ TYPE 2 MI, WITHIN 4 WKS OF PREVIOUS MI		I21.A1	**ACS**
12456194	TYPE 2 MI		I21.A1	**ACS**
12455814	OTHER MYOCARDIAL INFARCTION TYPE		I21.A9	**ACS**
12455813	MYOCARDIAL INFARCTION TYPE 2		I21.A1	**ACS**
12455812	ACUTE MYOCARDIAL INFARCTION UNSPECIFIED		I21.9	**ACS**
12455647	CAD W STABLE ANGINA		I25.118	**Stable CAD**

Internal DX_ID	Internal Diagnosis Description	ICD9 Code	ICD10 Code	ACS/Stable CAD
12198614	ST ELEVATION (STEMI) MYOCARDIAL INFARCTION OF UNSP SITE		I21.3	**ACS**
12194151	CORONARY ATHEROSCLEROSIS DUE TO CALCIFIED CORONARY LESION		I25.84	**Stable CAD**
12191095	ATHSCL HEART DISEASE OF NATIVE COR ART W UNSP ANG PCTRS		I25.119	**Stable CAD**
12190549	ATHSCL NATIVE COR ART OF TRANSPLANTED HEART W/O ANG PCTRS		I25.811	**Stable CAD**
12189452	ATHSCL HEART DISEASE OF NATIVE COR ART W UNSTABLE ANG PCTRS		I25.110	**Stable CAD**
12188884	ATHSCL NATIVE COR ART OF TXPLT HEART W UNSTABLE ANGINA		I25.750	**Stable CAD**
12188883	STEMI INVOLVING LEFT CIRCUMFLEX CORONARY ARTERY		I21.21	**ACS**
12181822	CORONARY ATHEROSCLEROSIS DUE TO LIPID RICH PLAQUE		I25.83	**Stable CAD**
12180280	NON-ST ELEVATION (NSTEMI) MYOCARDIAL INFARCTION		I21.4	**ACS**
12178928	OTHER FORMS OF CHRONIC ISCHEMIC HEART DISEASE		I25.89	**Stable CAD**
12177581	CHRONIC ISCHEMIC HEART DISEASE, UNSPECIFIED		I25.9	**Stable CAD**
12177580	OTHER FORMS OF ACUTE ISCHEMIC HEART DISEASE		I24.8	**ACS**
12176915	CHRONIC TOTAL OCCLUSION OF CORONARY ARTERY		I25.82	**Stable CAD**
12173939	ANGINA PECTORIS WITH DOCUMENTED SPASM		I20.1	**Stable CAD**
12170536	OTHER FORMS OF ANGINA PECTORIS		I20.8	**Stable CAD**
12169761	ANGINA PECTORIS, UNSPECIFIED		I20.9	**Stable CAD**
12165899	ATHSCL NONAUT BIOLOGICAL CABG W ANG PCTRS W DOCUMENTED SPASM		I25.731	**Stable CAD**
12164288	ATHSCL BYPASS OF COR ART OF TXPLT HEART W ANG PCTRS W SPASM		I25.761	**Stable CAD**
12155501	CAD WO ANGINA, W HX OF PERCUTANEOUS CORONARY ANGIOPLASTY	414.01, V45.82	I25.10, Z98.61	**Stable CAD**
12149198	HX OF NONISCHEMIC MI	412	I25.2	**Stable CAD**
12147867	CALCIFIC CORONARY ATHEROSCLEROSIS WO ANGINA	414.00, 414.4	I25.10, I25.84	**Stable CAD**

Internal DX_ID	Internal Diagnosis Description	ICD9 Code	ICD10 Code	ACS/Stable CAD
12147537	CORONARY ATHEROSCL DUE TO CALCIFIED CORON LESION	414.4		**Stable CAD**
12147109	ACUTE ST ELEVATION MI, LEFT MAIN CORONARY ARTERY	410.10	I21.01	**ACS**
12147108	ACUTE ST ELEVATION MI, LEFT ANTERIOR DESCENDING CORONARY ARTERY	410.10	I21.02	**ACS**
12147106	MI, ACUTE, ST SEGMENT ELEVATION, INITIAL EPISODE.	410.91	I21.3	**ACS**
12147104	MI, ACUTE, SEPTUM, INITIAL EPISODE.	410.81	I21.29	**ACS**
12147102	MI, ACUTE, POSTEROBASAL WALL, INITIAL EPISODE.	410.61	I21.29	**ACS**
12147100	MI, ACUTE, NON ST SEGMENT ELEVATION, INITIAL EPISODE.	410.71	I21.4	**ACS**
12147097	MI, ACUTE, ATRIUM, INITIAL EPISODE.	410.81	I21.29	**ACS**
12147096	MI, < 4WEEKS.	410.90	I21.3	**ACS**
12146951	MI, ACUTE, POSTEROLATERAL WALL.	410.50	I21.29	**ACS**
12146950	MI, ACUTE, ANTEROSEPTAL.	410.10	I21.09	**ACS**
12146938	HX OF ST ELEVATION MI	412	I25.2	**Stable CAD**
12146881	CAD WO ANGINA, TRIPLE VESSEL	414.0	I25.10	**Stable CAD**
12146880	CAD WO ANGINA, MULTIPLE VESSEL	414.0	I25.10	**Stable CAD**
12146879	CAD WO ANGINA, DOUBLE VESSEL	414.0	I25.10	**Stable CAD**
12146878	CAD OF AUTOLOGOUS BYPASS GRAFT, WO ANGINA	414.05	I25.810	**Stable CAD**
12146877	CAD OF AUTOLOGOUS ARTERIAL GRAFT, WO ANGINA	414.04	I25.810	**Stable CAD**
12145994	CAD WO ANGINA, W HX OF CABG	414.00, V45.81	I25.10, Z95.1	**Stable CAD**
12144236	CAD WO ANGINA DUE TO LIPID RICH PLAQUE	414.00, 414.3	I25.10, I25.83	**Stable CAD**
12143070	CHRONIC TOTAL OCCLUSION OF CORONARY ARTERY, WO ANGINA	414.00, 414.2	I25.10, I25.82	**Stable CAD**
12142645	CHRONIC TOTAL OCCLUSION OF CORONARY ARTERY	414.2		**Stable CAD**

Internal DX_ID	Internal Diagnosis Description	ICD9 Code	ICD10 Code	ACS/Stable CAD
12142342	MYOCARDIAL INFARCTION (MI), ACUTE, ANTEROLATERAL WALL < = 8 WKS.	410.0		**ACS**
12142341	MYOCARDIAL INFARCTION (MI), ACUTE, INFEROLATERAL WALL < = 8 WKS.	410.20		**ACS**
12142340	MYOCARDIAL INFARCTION (MI), ACUTE, INFERIOR WALL < = 8 WKS.	410.40		**ACS**
12142339	MYOCARDIAL INFARCTION (MI), ACUTE, LATERAL WALL < = 8 WKS.	410.50		**ACS**
12142338	MYOCARDIAL INFARCTION (MI), ACUTE, TRUE POSTERIOR WALL < = 8 WK.	410.60		**ACS**
12142337	MYOCARDIAL INFARCTION (MI), ACUTE, SUBENDOCARDIAL < = 8 WKS.	410.70		**ACS**
12142336	MYOCARDIAL INFARCTION (MI), ACUTE, ANTERIOR WALL < = 8 WKS.	410.10		**ACS**
12142136	CAD WO ANGINA, W HX OF OLD MI WO HX OF CABG	414.00, 412	I25.10, I25.2	**Stable CAD**
12141664	CAD OF INTERNAL MAMMARY ARTERY GRAFT, WO ANGINA	414.04	I25.810	**Stable CAD**
12141559	CAD WO ANGINA, POST PERCUTANEOUS CORONARY ANGIOPLASTY	414.00, V45.82	I25.10, Z98.61	**Stable CAD**
12141558	CAD WO ANGINA, W HX OF MI	414.00, 412	I25.10, I25.2	**Stable CAD**
12141557	CAD WO ANGINA, PRESENCE OF STENT	414.00, V45.82	I25.10, Z95.5	**Stable CAD**
12140557	MYOCARDIAL INFARCTION, NON Q WAVE.	410.90		**ACS**
12140556	MYOCARDIAL INFARCTION.	410.90		**ACS**
12140244	ACUTE NON ST ELEVATION MI	410.70	I21.4	**ACS**
12140227	HX OF NON ST ELEVATION MI	412	I25.2	**Stable CAD**
12140201	CARDIOMYOPATHY, DILATED, ISCHEMIC.	414.8		**Stable CAD**
12139924	ACUTE ST ELEVATION MI	410.90	I21.3	**ACS**
12139251	CARDIAC SYNDROME X	413.9	I20.9	**Stable CAD**
12139245	MYOCARDIAL INFARCT, OLD (>8 WEEKS).	412		**Stable CAD**
12138468	CORONARY ATHEROSCLEROSIS.	414.0		**Stable CAD**

Internal DX_ID	Internal Diagnosis Description	ICD9 Code	ICD10 Code	ACS/Stable CAD
12137993	MYOCARDIAL INFARCT NOS.	410.90		**ACS**
12137992	SUBENDOCARDIAL INFARCT.	410.70		**ACS**
12137991	AMI LATERAL WALL NEC.	410.50		**ACS**
12137990	AMI INFERIOR WALL NEC.	410.40		**ACS**
12137989	AMI INFEROLATERAL WALL.	410.20		**ACS**
12137988	AMI ANTEROLATERAL WALL.	410.0		**ACS**
12137987	ACUTE MYOCARDIAL INFARCTION.	410.0		**ACS**
12137451	STABLE ANGINA	413.9	I20.8	**Stable CAD**
12135512	CAD OF BYPASS GRAFT OF TRANSPLANTED HEART, WO ANGINA	414.07	I25.812	**Stable CAD**
12135340	CHRONIC CORONARY INSUFFICIENCY	414.8	I25.89	**Stable CAD**
12135338	ACUTE MI, ANTERIOR WALL.	410.10		**ACS**
12135071	HX OF MYOCARDIAL INFARCTION, CAD.	412		**Stable CAD**
12133320	CAD IN CABG.	414.0		**Stable CAD**
12133319	CAD AFTER PERCUTANEOUS TRANSLUMINAL ANGIOPLASTY.	414.01		**Stable CAD**
12133208	CAD WO ANGINA, RADIATION INDUCED	414.00, E926.9	I25.10, Y84.2	**Stable CAD**
12133125	MYOCARDIAL INFARCT: HISTORY OF: >8 WEEKS.	412		**Stable CAD**
12132434	CORONARY ARTERY DISEASE (CAD).	414.01		**Stable CAD**
12132155	CAD S/P CORONARY ARTERY BY PASS GRAFT.	414.05		**Stable CAD**
12131828	ANGINA PECTORIS, STABLE.	413.9		**Stable CAD**
12131827	MI, ACUTE, W RUPTURE OF VENTRICLE.	410.90		**ACS**
12130089	HX OF MYOCARDIAL INFARCTION (MI), >8 WEEKS.	412		**Stable CAD**

Internal DX_ID	Internal Diagnosis Description	ICD9 Code	ICD10 Code	ACS/Stable CAD
12130088	HX OF MYOCARDIAL INFARCTION (MI).	410.90		**ACS**
12129243	ANGINA, STABLE.	413.9		**Stable CAD**
12129146	MYOCARDIAL INFARCTION	410.90		**ACS**
12129134	MI DUE TO THROMBOSIS OF LEFT ANTERIOR DESCENDING CORONARY ARTERY	410.90		**ACS**
12127479	MI, ACUTE, SUBSEQUENT EPISODE OF CARE..	410.82		**ACS**
12127478	MI, ACUTE, LATERAL WALL, INITIAL EPISODE.	410.51	I21.29	**ACS**
12127203	CAD IN CABG, INTERNAL MAMMARY ARTERY.	414.0		**Stable CAD**
12126772	CAD, SINGLE VESSEL WO ANGINA	414.0	I25.10	**Stable CAD**
12126018	MI, ACUTE, SUBENDOCARDIAL.	410.70		**ACS**
12125179	CAD OF NATIVE ARTERY OF TRANSPLANTED HEART, WO ANGINA	414.06	I25.811	**Stable CAD**
12125073	NOCTURNAL ANGINA	413	I20.8	**Stable CAD**
12123820	CAD OF CABG WO ANGINA	414.05	I25.810	**Stable CAD**
12123819	CAD OF ARTERIAL BYPASS GRAFT.	414.04		**Stable CAD**
12123235	CAD OF NATIVE ARTERY WO ANGINA	414.01	I25.10	**Stable CAD**
12123234	CAD OF NONAUTOLOGOUS GRAFT, WO ANGINA	414.03	I25.810	**Stable CAD**
12123233	CAD OF AUTOLOGOUS VEIN GRAFT, WO ANGINA	414.02	I25.810	**Stable CAD**
12123191	ARTERIOSCLEROTIC CARDIOVASCULAR DISEASE WO ANGINA	429.2, 414.00	I25.10	**Stable CAD**
12123081	MI, ACUTE.	410.80		**ACS**
12123080	MI, ACUTE, LATERAL WALL.	410.50		**ACS**
12123079	MI, ACUTE, INFERIOR WALL.	410.40		**ACS**
12123077	MI, ACUTE, ANTERIOR WALL.	410.10		**ACS**

Internal DX_ID	Internal Diagnosis Description	ICD9 Code	ICD10 Code	ACS/Stable CAD
12123002	MI, ACUTE, INITIAL EPISODE.	410.91	I21.3	**ACS**
12020726	CHRONIC ISCHEMIC HEART DISEASE	414.9	I25.9	**Stable CAD**
12020725	CORONARY ATHEROSCLEROSIS.	414.0		**Stable CAD**
12020723	ACUTE MI	410.90	I21.9	**ACS**
12019534	ANGINA, UNSPECIFIED	413.9	I20.9	**Stable CAD**
12019533	MI, < 8 WEEKS.	410.90	I21.3	**ACS**
12019532	MI, ACUTE, LATERAL WALL.	410.50	I21.29	**ACS**
12019531	MI, ACUTE, INFERIOR WALL.	410.40	I21.19	**ACS**
12019530	MI, ACUTE, INFEROPOSTERIOR WALL.	410.30	I21.19	**ACS**
12019529	MI, ACUTE, INFEROLATERAL WALL.	410.20		**ACS**
12019528	MI, ACUTE, ANTERIOR WALL.	410.10	I21.09	**ACS**
12019527	MI, ACUTE, ANTEROLATERAL WALL.	410.0	I21.09	**ACS**
12018783	PRINZMETAL ANGINA	413.1	I20.1	**Stable CAD**
12018322	ANGINA DECUBITUS	413	I20.8	**Stable CAD**
12018321	HX OF MI.	412	I25.2	**Stable CAD**
12017574	FIRST MYOCARDIAL INFARCTION	410.91		**ACS**
12017071	MI, ACUTE, SUBENDOCARDIAL..	410.70		**ACS**
12015982	MI, ACUTE, LEFT CORONARY ARTERY.	410.90	I21.3	**ACS**
12015981	MI, ACUTE, SEPTUM.	410.80	I21.29	**ACS**
12015980	MI, ACUTE, RIGHT CORONARY ARTERY.	410.90	I21.11	**ACS**
12015979	MI, ACUTE, ATRIUM.	410.80	I21.29	**ACS**

Internal DX_ID	Internal Diagnosis Description	ICD9 Code	ICD10 Code	ACS/Stable CAD
12015978	ACUTE INFARCTION OF PAPILLARY MUSCLE.	410.80		**ACS**
12015973	MI, ACUTE, POSTERIOR WALL, INITIAL EPISODE.	410.61	I21.29	**ACS**
12015972	MI, ACUTE, POSTERIOR WALL.	410.60	I21.29	**ACS**
12015971	MI, ACUTE, POSTEROBASAL WALL.	410.60	I21.29	**ACS**
12015969	MI, ACUTE, POSTEROLATERAL WALL, INITIAL EPISODE.	410.51	I21.29	**ACS**
12015967	MI, ACUTE, INFERIOR WALL, INITIAL EPISODE.	410.41	I21.19	**ACS**
12015965	MI, ACUTE, INFEROPOSTERIOR WALL, INITIAL EPISODE.	410.31	I21.19	**ACS**
12015963	MI, ACUTE, INFEROLATERAL WALL, INITIAL EPISODE.	410.21	I21.19	**ACS**
12015961	MI, ACUTE, ANTEROSEPTAL, INITIAL EPISODE OF CARE.	410.11		**ACS**
12015959	MI, ACUTE, ANTEROLATERAL WALL, INITIAL EPISODE.	410.01	I21.09	**ACS**
12015161	ISCHEMIC CARDIOMYOPATHY	414.8	I25.5	**Stable CAD**
12015156	CAD (CORONARY ARTERY DISEASE) WO ANGINA	414.0	I25.10	**Stable CAD**
12014258	CHRONIC STABLE ANGINA.	413.9		**Stable CAD**
1904140	ARTERIOSCLEROTIC HEART DISEASE	414.0		**Stable CAD**
1901652	AMI ANTEROLATERAL WALL	410.10		**ACS**
1901651	ACUTE MYOCARDIAL INFARCT	410.0		**ACS**
1207214	OTHER SPEC FORMS CHRONIC ISCHEMIC HEART DISEASE	414.8		**Stable CAD**
1207210	CORONARY ATHEROSLERO UNSPEC TYPE BYPASS GRAFT	414.05		**Stable CAD**
1207209	CORONARY ATHEROSCLEROSIS OF ARTERY BYPASS GRAFT	414.04		**Stable CAD**
1207208	COR ATHEROSLERO NONAUTOL BIOLOGICAL BYPS GRAFT	414.03		**Stable CAD**
1207207	CORONARY ATHEROSLERO AUTOL VEIN BYPASS GRAFT	414.02		**Stable CAD**

Internal DX_ID	Internal Diagnosis Description	ICD9 Code	ICD10 Code	ACS/Stable CAD
1207206	CORONARY ATHEROSCLEROSIS NATIVE CORONARY ARTERY	414.01		**Stable CAD**
1207205	PRINZMETAL ANGINA	413.1		**Stable CAD**
1207202	ACUTE MYOCARD INFARCT UNSPEC SITE INIT EPIS CARE	410.91		**ACS**
1207201	ACUT MYOCARD INFARCT OTH SITE SUBSQT EPIS CARE	410.82		**ACS**
1207200	ACUT MYOCARD INFARCT OTH SITE INIT EPIS CARE	410.81		**ACS**
1207199	ACUT MYOCARD INFARCT OTH SPEC SITE EPIS CARE UNS	410.80		**ACS**
1207197	ACUT MI SUBENDOCARDIAL INFARCT EPIS CARE UNS	410.70		**ACS**
1207196	ACUT MI TRUE POST WALL INFARCT SUBSQT EPIS CARE	410.62		**ACS**
1207195	ACUT MI TRUE POST WALL INFARCT INIT EPIS CARE	410.61		**ACS**
1207194	ACUT MI TRUE POST WALL INFARCT EPIS CARE UNS	410.60		**ACS**
1207193	ACUT MI OTH LAT WALL SUBSQT EPIS CARE	410.52		**ACS**
1207192	ACUT MYOCARD INFARCT OTH LAT WALL INIT EPIS CARE	410.51		**ACS**
1207191	ACUT MI OTH INF WALL SUBSQT EPIS CARE	410.42		**ACS**
1207190	ACUT MYOCARD INFARCT OTH INF WALL INIT EPIS CARE	410.41		**ACS**
1207189	ACUT MI INFEROPOST WALL SUBSQT EPIS CARE	410.32		**ACS**
1207188	ACUT MI INFEROPOST WALL INIT EPIS CARE	410.31		**ACS**
1207187	ACUT MI INFEROLAT WALL SUBSQT EPIS CARE	410.22		**ACS**
1207186	ACUT MI INFEROLAT WALL INIT EPIS CARE	410.21		**ACS**
1207185	ACUT MI OTH ANT WALL SUBSQT EPIS CARE	410.12		**ACS**
1207184	ACUT MYOCARD INFARCT OTH ANT WALL INIT EPIS CARE	410.11		**ACS**
1207183	ACUT MI ANTEROLAT WALL SUBSQT EPIS CARE	410.02		**ACS**

Internal DX_ID	Internal Diagnosis Description	ICD9 Code	ICD10 Code	ACS/Stable CAD
1207182	ACUT MI ANTEROLAT WALL INIT EPIS CARE	410.01		**ACS**
1203518	ACUT MI SUBENDOCARDIAL INFARCT INIT EPIS CARE	410.71	I21.4	**ACS**
1202907	UNSPECIFIED CHRONIC ISCHEMIC HEART DISEASE	414.9		**Stable CAD**
1202906	COR ATHEROSLERO UNSPEC TYPE VESSEL NATIVE/GRAFT	414.0		**Stable CAD**
1202904	ACUT MYOCARD INFARCT UNS SITE EPIS CARE UNS	410.90		**ACS**
120923	OTHER AND UNSPECIFIED ANGINA PECTORIS	413.9		**Stable CAD**
120922	ANGINA DECUBITUS	413		**Stable CAD**
120921	OLD MYOCARDIAL INFARCTION	412		**Stable CAD**
120919	ACUT MYOCARD INFARCT UNS SITE SUBSQT EPIS CARE	410.92		**ACS**
120918	ACUT MYOCARD INFARCT OTH LAT WALL EPIS CARE UNS	410.50		**ACS**
120917	ACUT MYOCARD INFARCT OTH INF WALL EPIS CARE UNS	410.40		**ACS**
120916	ACUT MI INFEROPOST WALL EPIS CARE UNS	410.30		**ACS**
120915	ACUT MI INFEROLAT WALL EPIS CARE UNS	410.20		**ACS**
120914	ACUT MYOCARD INFARCT OTH ANT WALL EPIS CARE UNS	410.10		**ACS**
120913	ACUT MI ANTEROLAT WALL EPIS CARE UNS	410.0		**ACS**

Supplemental Figure 1. Flow Diagram of Patient Entry into Study

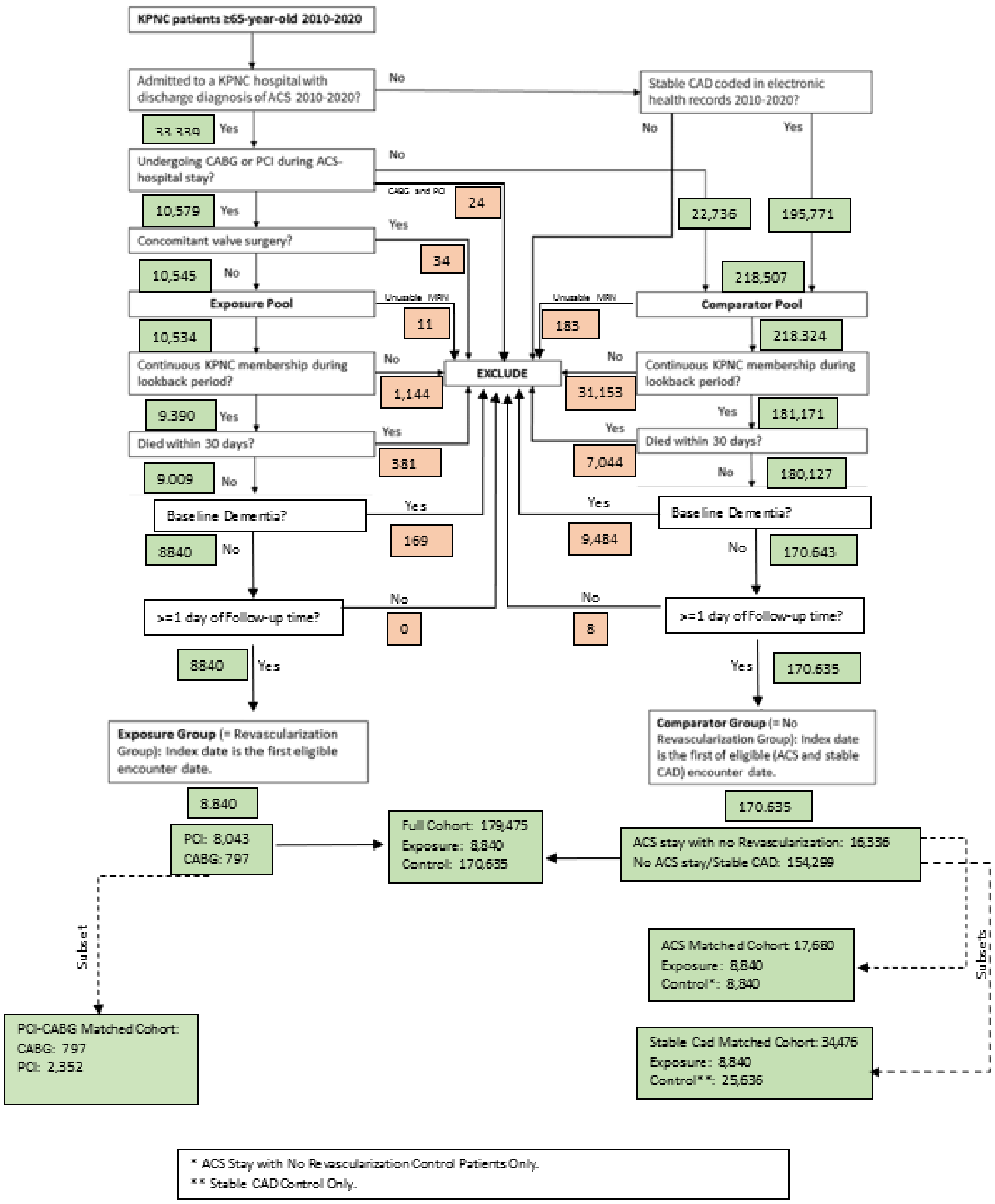

Supplementary Table 2. Internal Diagnosis codes used to identify Dementia.

Note: Kaiser Permanente codes diagnoses using an internal coding system that maps to ICD9/10 codes. The below table shows the internal codes ("dx_id") that were used to identify Dementia.

Internal DX_ID	Internal Diagnosis Description	Dementia Type	ICD9 Code	ICD10 Code
12127408	LEWY BODY DEMENTIA	Lewy Body/Parkinsons	331.82, 294.10	G31.83, F02.80
12123903	DEMENTIA IN PARKINSONS DISEASE.	Lewy Body/Parkinsons	331.82, 294.10	G20, F02.80
12135686	FRONTOTEMPORAL DEMENTIA.	FTD	331.19, 294.10	G31.09, F02.80
12123900	DEMENTIA DUE TO PICKS DISEASE	Other	331.11, 294.10	G31.01, F02.80
12020651	MULTI INFARCT DEMENTIA, UNCOMPLICATED.	Other	290.40, 437.0	F01.50
12141644	MULTI INFARCT DEMENTIA	Vascular	290.40, 437.0	F01.50
12141742	VASCULAR DEMENTIA	Vascular	290.40, 437.0	F01.50
12147206	VASCULAR DEMENTIA, UNCOMPLICATED.	Vascular	290.40, 437.0	F01.50
12141757	DEMENTIA DUE TO CREUTZFELDT JAKOB DISEASE	Other	046.19, 294.10	A81.00, F02.80
12141396	LEWY BODY DISEASE	Lewy Body/Parkinsons	331.82	G31.83, F02.80
12018012	DEMENTIA WITH LEWY BODIES	Lewy Body/Parkinsons	331.82	
12137485	DIFFUSE LEWY BODY DISEASE.	Lewy Body/Parkinsons	331.82	
12018011	OTHER FRONTOTEMPORAL DEMENTIA	FTD	331.19	
12139076	DEMENTIA D/T PICK'S DISEASE.	Other	331.11	
12136995	FRONTOTEMPORAL DEMENTIA.	FTD	331.1	
12018193	ALZHEIMERS DISEASE	Alzheimers	331.0	G30.9, F02.80
12147666	ALZHEIMERS DISEASE, LATE ONSET	Alzheimers	331.0	G30.1, F02.80
12147665	ALZHEIMERS DISEASE, EARLY ONSET	Alzheimers	331.0	G30.0, F02.80

Internal DX_ID	Internal Diagnosis Description	Dementia Type	ICD9 Code	ICD10 Code
120532	ALZHEIMERS DISEASE	Alzheimers	331.0	
12129636	DEMENTIA, ALZHEIMER'S TYPE, EARLY ONSET.	Alzheimers	331.0	
12129637	DEMENTIA, ALZHEIMER'S TYPE, LATE ONSET.	Alzheimers	331.0	
12017075	ORGANIC DEMENTIA	Alzheimers	294.8	F03.90
12129640	MILD DEMENTIA	Unspecified	294.8	F03.90
12129641	MODERATE DEMENTIA	Unspecified	294.8	F03.90
12129642	SEVERE DEMENTIA	Unspecified	294.8	F03.90
12135678	MIXED DEMENTIA	Unspecified	294.8	F03.90
12129633	DEMENTIA D/T HEAD TRAUMA.	Other	294.8	
12129643	DEMENTIA, UNSPECIFIED.	Other	294.8	
12135679	FRONTOTEMPORAL DEMENTIA.	FTD	294.8	
12015082	DEMENTIA	Alzheimers	294.2	F03.90
121213370	MAJOR NEUROCOGNITIVE DISORDER DUE TO MULTIPLE ETIOLOGIES WO BEHAVIORAL DISTURBANCE	Alzheimers	294.1	F03.90
121213367	MAJOR NEUROCOGNITIVE DISORDER DUE TO ANOTHER MEDICAL CONDITION WO BEHAVIORAL DISTURBANCE	Alzheimers	294.1	F02.80
12017042	DEMENTIA DUE TO A GENERAL MEDICAL CONDITION.	Other	294.1	
12123905	DEMENTIA, DUE TO CREUTZFELDT JAKOB DISEASE.	Other	294.1	
12123906	DEMENTIA, DUE TO HUNTINGTONS DISEASE.	Other	294.1	
12125648	PRIMARY DEGENERATIVE DEMENTIA OF ALZHEIMER TYPE, SENILE ONSET, UNCOMPLICATED.	Alzheimers	294.1	
12129634	DEMENTIA, AIDS COMPLEX RELATED	Other	294.1	
12129635	DEMENTIA, AIDS RELATED.	Other	294.1	

Internal DX_ID	Internal Diagnosis Description	Dementia Type	ICD9 Code	ICD10 Code
12129638	DEMENTIA DUE TO PARKINSON'S DISEASE.	Lewy Body/Parkinsons	294.1	
12129639	DEMENTIA, D/T PICK'S DISEASE.	Other	294.1	
12136604	PRIMARY DEGENERATIVE DEMENTIA	Alzheimers	290.9	F03.90
12124959	MULTI INFARCT DEMENTIA.	Vascular	290.4	
12129644	DEMENTIA, VASCULAR..	Vascular	290.4	
12132247	VASCULAR DEMENTIA, UNCOMPLICATED.	Vascular	290.4	
12136964	VASCULAR DEMENTIA.	Vascular	290.4	
12138450	VASCULAR DEMENTIA.	Vascular	290.4	
12142120	UNCOMPLICATED VASCULAR DEMENTIA.	Vascular	290.4	
12019167	SENILE DEMENTIA-	Other	290.0	F03.90
12168742	DEMENTIA WITH LEWY BODIES	Lewy Body/Parkinsons		G31.83
12170052	OTHER FRONTOTEMPORAL DEMENTIA	FTD		G31.09
12171391	ALZHEIMER'S DISEASE, UNSPECIFIED	Alzheimers		G30.9
12168741	OTHER ALZHEIMER'S DISEASE	Alzheimers		G30.8
12172781	ALZHEIMER'S DISEASE WITH LATE ONSET	Alzheimers		G30.1
12173310	ALZHEIMER'S DISEASE WITH EARLY ONSET	Alzheimers		G30.0

Supplementary Table 3. Comorbidities and Corresponding ICD-9 and ICD-10 Diagnosis Codes Used for Covariate Definition

Comorbidity	Corresponding ICD-9 Codes	Corresponding ICD-10 Codes
Anemia	280, 281, 282, 283, 284, 285	D50, D51, D52, D53, D55, D56, D57, D58, D59, D60, D61, D62, D63, D64
Atrial fibrillation	427.3	I48
Cerebrovascular disease	430, 431, 432, 433, 434, 435, 437.0, 437.1, 437.7, 437.8, 362.34	I60, I61, I62, I63, I65, G45, G46
Chronic kidney disease	585.3, 583.4, 585.5	N18.3, N18.4, N18.5
Chronic liver disease	570, 571, 572, 573	K70, K71, K72, K73, K74, K75, K76, K77
Chronic lung disease	490, 491, 492, 493, 494, 495, 496	J40-J47
Coronary artery disease	410, 411, 412, 413, 414, V45.81, V45.82, V45.09	I20, I25, Z95.1, Z95.5, Z98.61, I21, I22, I23, I24, Z95.818
Depression	296.2, 296.3	F32, F33
Diabetes	250	E08, E09, E10, E11, E13
Heart failure/cardiomyopathy	425, 428	I42, I43, I50
Hyperlipidemia	272	E78.0, E78.1, E78.2, E78.3, E78.4, E78.5, E78.6
Hypertension	401.0, 401.1, 401.9	I10, I11, I12, I13
Known clotting disorders	286, 287	D66, D67, D68, D69
Malignancy	140-208, 230-239	C00-C96
Non-cerebrovascular chronic neurologic disease	331, 332, 333, 334, 335, 336	G20, G21, G23, G24, G25, G26, G30, G31, G32
Peripheral vascular disease	440, 441, 442, 443.9	I70, I71, I72
Presence of permanent pacemaker	V45.01, V45.02	Z95.0, Z95.810
Sleep apnea	780.57	G47.3
Valvular heart disease	424, V42.2, V43.3	I34.0, I35.0, I35.1, I36.1

Supplementary Table 4. Power Analysis

We hypothesized that revascularization (either PCI or CABG) will have the same risk of dementia in older adults aged 65 and older with ACS compared to patients with either stable CAD or ACS without revascularization. The expected rate of overall new dementia in the 'stable CAD or ACS without revascularization' is 7% and the absolute margin is 1.5%. The equivalence test based on normal approximation calculation method for the difference of two proportions to estimate the power calculation was used. When the number of subjects in the treatment group ('ACS with PCI/CABG' group) is 3309 and the number of subjects in the reference group ('stable CAD or ACS without PCI/CABG' group) is 9927, a Z-test (Unpooled) has 80% power to reject the null hypothesis that the difference in the response rate in the treatment group and the response rate in the reference group is outside the margin of equivalence of -0.015 to 0.015, i.e. that the treatment and reference are not equivalent, in favor of the alternative hypothesis that the response rates of the treatment and reference groups are equivalent, assuming that the actual difference in response rates is 0, the response rate in the reference group is 0.07, and that the test is made at the 5% significance level. Our feasibility study result showed we would have at least 4000 patients in the 'ACS with PCI/CABG' group and at least 12,000 in the 'stable CAD or ACS without PCI/CABG' group; therefore, the power will reach 88%.

Supplementary Table 5. Propensity Score Matching Methods

a. General Method

i. Calculation of propensity scores: Propensity scores were calculated using logistic regression predicting treatment status (i.e. exposure vs. control) using patient baseline characteristics to attain the best fit. See details as to which variables were used for which matched cohort in the table below.

ii. Selection of matched controls: For every exposure patient, 1 to 3 (see table below for specifics per cohort) matched control patients were selected for the propensity-matched cohort using the propensity scores calculated in [i] and using the greedy method.

iii. Assessment of balance in matched cohort: Covariate balance between treatment groups after propensity score matching was assessed using standardized mean differences, with values less than 0.10 indicating adequate balance.

b. Summary of methods/balance diagnostics for three matched cohorts:

Cohort	Covariates used to calculate propensity score	Number of matched controls selected	Standardized mean difference of Matched cohort
Exposure: (PCI or CABG) Control: ACS with no Revascularization.	Age<br>Sex<br>Race/Ethnicity<br>NDI<br>BMI<br>Smoking Status<br>Elixhauser Comorbidity Score	1	.0042
Exposure: (PCI or CABG) Control: Stable CAD	Age<br>Sex<br>Race/Ethnicity<br>NDI<br>BMI<br>Hypertension<br>Diabetes<br>Hyperlipidemia<br>Cerebrovascular Disease<br>Peripheral Vascular Disease<br>Heart Failure<br>Cardiomyopathy<br>Valvular Heart Disease<br>Atrial Fibrillation<br>Malignancy<br>Anemia<br>Chronic Lung Disease<br>Sleep Apnea<br>Chronic Liver Disease<br>Chronic Kidney Disease	1-3	.0192

	Non-cerebrovascular chronic neurologic disease<br>Depression<br>Smoking Status<br>Elixhauser comorbidity score<br>Creatinine<br>Hemoglobin<br>Platelets<br>WBC<br>Glucose<br>HbA1c<br>eGFR<br>Cholesterol<br>HDL<br>Triglycerides<br>BNP<br>Ejection Fraction<br>Antiplatelet Medication<br>Statin Medication<br>Other Cardiovascular Medication		
Exposure: CABG<br>Control: Stable CAD	Age<br>Sex<br>Race/Ethnicity<br>NDI<br>BMI<br>Diabetes<br>Hiperlipidemia<br>Cerebrovascular Disease<br>Peripheral Vascular Disease<br>Valvular Heart Disease<br>Atrial Fibrillation<br>Malignancy<br>Anemia<br>Chronic Lung Disease<br>Sleep Apnea<br>Non-cerebrovascular chronic neurologic disease<br>Depression<br>Smoking Status<br>Elixhauser comorbidity score<br>Creatinine<br>Hemoglobin<br>Platelets<br>WBC<br>Glucose<br>HbA1c<br>eGFR<br>Cholesterol<br>HDL<br>Triglycerides<br>Antiplatelet Medication<br>Statin Medication<br>Other Cardiovascular Medication	1-3	.1084

Supplementary Figure 1. Kaplan-Meier Unadjusted Probability Curves for Incident Dementia of Matched Cohorts after 1-Year Washout: CABG vs PCI

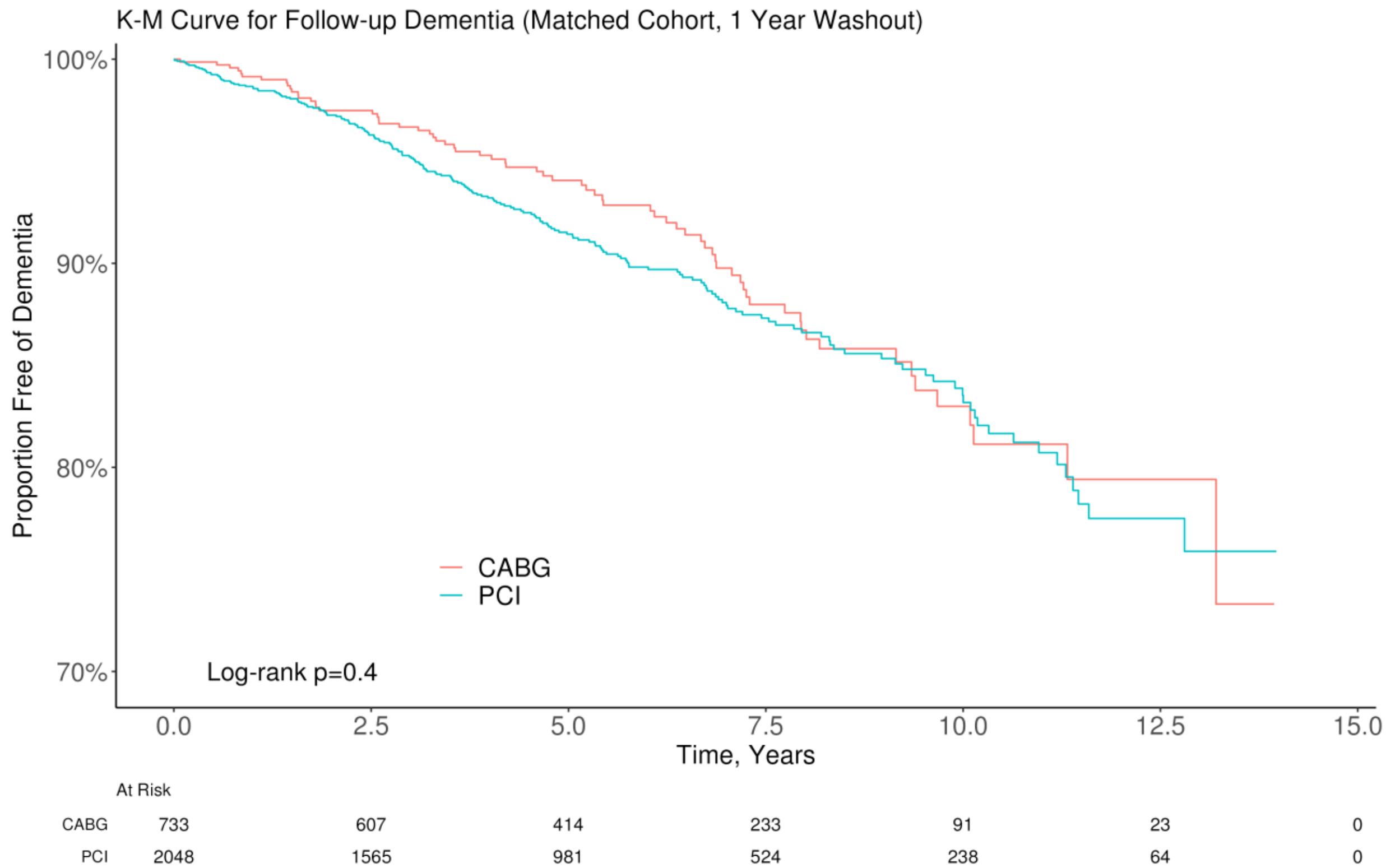

Supplementary Figure 2. KM Curve for Incident Dementia: Exposure (ACS with revascularization) vs. ACS with No Revascularization. Matched Cohort, No Washout

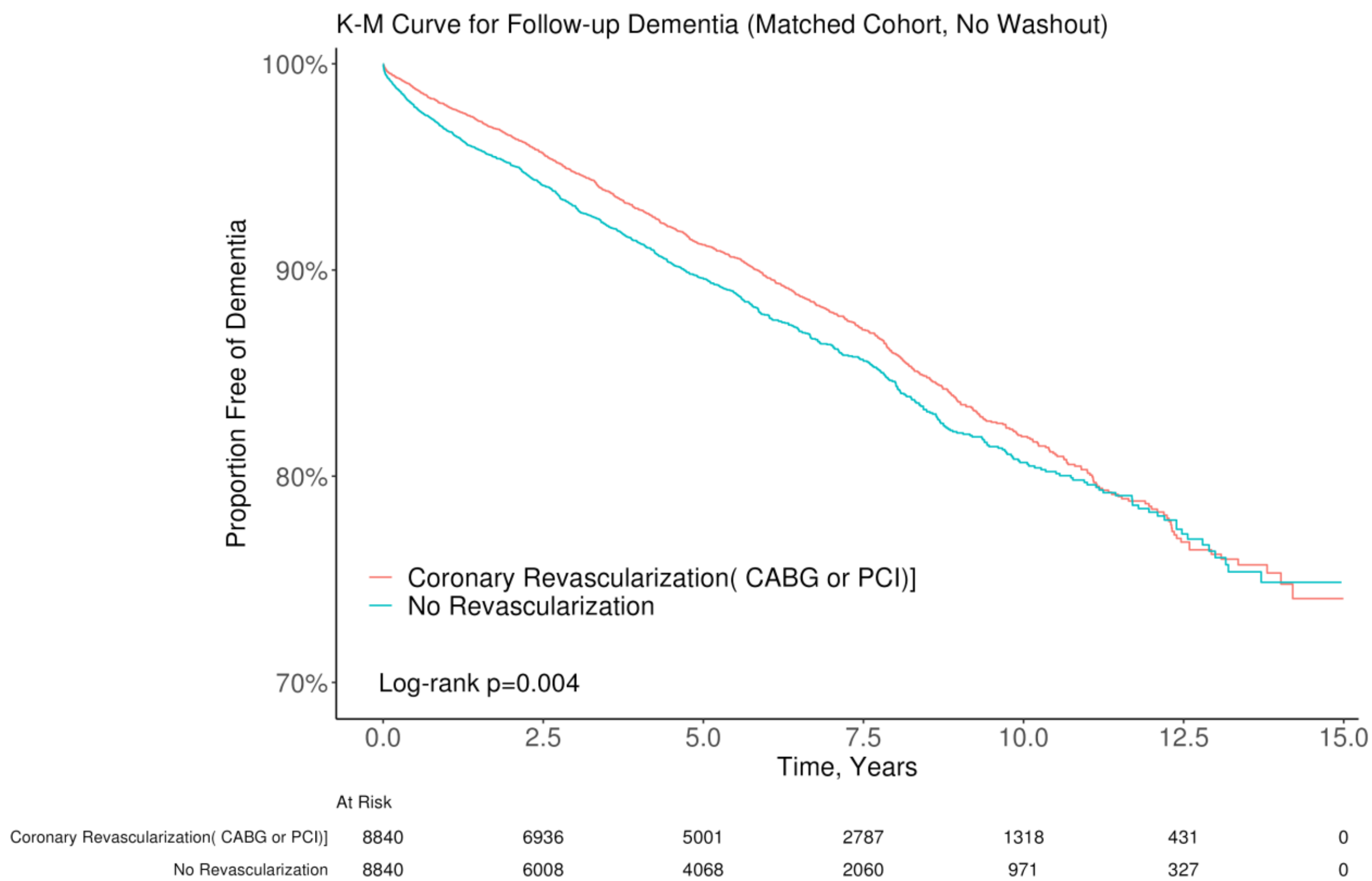

Supplement: Supplementary file 1 [file mmc1.pdf]
